# Supplementary material for: Integrating 3D genomic and epigenomic data to enhance target gene discovery and drug repurposing in transcriptome-wide association studies
Source: Nat Commun. 2022 Jun 7;13:3258. doi: 10.1038/s41467-022-30956-7 (PMC9171100; doi:10.1038/s41467-022-30956-7)
Supplement: Supplementary file 1 — Supplementary Information [file 41467_2022_30956_MOESM1_ESM.pdf]

# Supplementary Information for

## Integrating 3D genomic and epigenomic data to enhance target gene discovery and drug repurposing in transcriptome-wide association studies


**Authors:**
Chachrit Khunsriraksakul[1,2], Daniel McGuire[2,3], Renan Sauteraud[2,3], Fang Chen[2,3], Lina Yang[2,3], Lida Wang[2,3], Jordan Hughey[1,2], Scott Eckert[1,2], J. Dylan Weissenkampen[2,3], Ganesh Shenoy[4], Olivia Marx[5], Laura Carrel[6], Bibo Jiang[3,*], Dajiang J. Liu[1,2,3,*]

**1** Bioinformatics and Genomics Graduate Program; Pennsylvania State University College of Medicine; Hershey, Pennsylvania, 17033; USA.
**2** Institute for Personalized Medicine; Pennsylvania State University College of Medicine; Hershey, Pennsylvania, 17033; USA.
**3** Department of Public Health Sciences; Pennsylvania State University College of Medicine; Hershey, Pennsylvania, 17033; USA.
**4** Department of Neurosurgery; Pennsylvania State University College of Medicine; Hershey, Pennsylvania, 17033; USA.
**5** Biomedical Science Program; Pennsylvania State University College of Medicine; Hershey, Pennsylvania, 17033; USA.
**6** Department of Biochemistry and Molecular Biology; Pennsylvania State University College of Medicine; Hershey, Pennsylvania, 17033; USA.

**Manuscript correspondence should be addressed to**
Bibo Jiang: bjiang@phs.psu.edu
Dajiang J. Liu: dajiang.liu@psu.edu


**This file includes:**

Supplementary Notes
Supplementary Figures 1 to 13
Supplementary References

## Supplementary Notes

### ADMIXTURE analysis

To systemically identify European samples in Genotype-Tissue Expression project (GTEx)[1], Depression Gene Network (DGN)[2], and Common Mind Consortium (CMC)[3] cohorts, we utilized ADMIXTURE[4] to determine the ancestry fractions of each individual sample. We used 1000G Phase 3 reference panel that were derived from 5 major populations, including East Asian (EAS), South Asian (SAS), European (EUR), African (AFR), and Admixed (AMR) with a total sample size of 2,504[5]. It should be noted that European samples from Genetic European Variation in Disease (GEUVADIS)[6] can be determined directly from 1000 Genome reference panel. Supervised learning mode were applied to ~275,000 variants of each sample to make a prediction of the ancestry fractions of each sample (--supervised with K = 5 populations). The results indicated that 527 GTEx samples (out of 635), 427 CMC samples (out of 621), 873 DGN samples (out of 922) have European ancestry fraction > 0.90, and were retained for subsequent analysis. To confirm the validity of ADMIXTURE prediction, we plotted principal components of each cohort. Individuals with the same ADMIXTURE ancestry assignment were clustered together in the plot, which verified the accuracy of ancestry assignment (Supplementary Fig. 13).

### Genotype imputation

For genotype data derived from arrays (including CMC and DGN), SNPs with MAF > 0.05, Hardy-Weinberg equilibrium p-value > 0.05, and non-ambiguous alleles are used for imputation on the University of Michigan Imputation Server. We only retained imputed SNPs with MAF > 0.05 and imputation $R^2 > 0.8$ for subsequent analysis. We then created an allelic dosage file (counts of reference allele as appears on hg19 reference genome) via PLINK1.9 (--recode A-transpose)[7].

### Implementation of previous TWAS methods to create gene expression prediction models

For FUSION, we followed its original implementation[8]. Specifically, GEMMA[9, 10] was applied with the following parameters: missingness threshold (-miss) = 1, MAF threshold (-maf) = 0, r-squared threshold (-r2) = 1, recording pace (-rpace) = 1000, writing pace (-wpace) = 1000, analysis choice (-bslmm) = 2 for GBLUP.

For TIGAR, we used default settings in DPR software[11, 12]. We applied variational Bayesian algorithm. Specifically, DPR_VB was applied with the following parameters: model (-dpr) = 1.

For EpiXcan, we obtained pre-computed penalty factors from EpiXcan's online repository[13]. It should be noted that there are no pre-computed penalty factors for lymphoblastoid cell line; therefore, we used whole blood's penalty factors as proxy. Next, we performed elastic net regularization (using R package glmnet[14]) using pre-computed penalty factor.

For UTMOST, we followed CTIMP framework with default settings[15].

## Supplementary Figures

**Supplementary Figure 1: Comparison of median Spearman's rank correlation coefficient in simulated external dataset**. In panel **a**, PUMICE is compared to other single-tissue TWAS methods (i.e., PrediXcan, FUSION, TIGAR, and EpiXcan). In panel **b**, PUMICE is compared to UTMOST across different combinations of genetic correlation between causal and correlated tissues ($\rho$) and number of correlated tissues ($N_{corr}$). PUMICE consistently outperforms all single tissue methods and outperforms UTMOST when the training sample sizes are greater than 100.

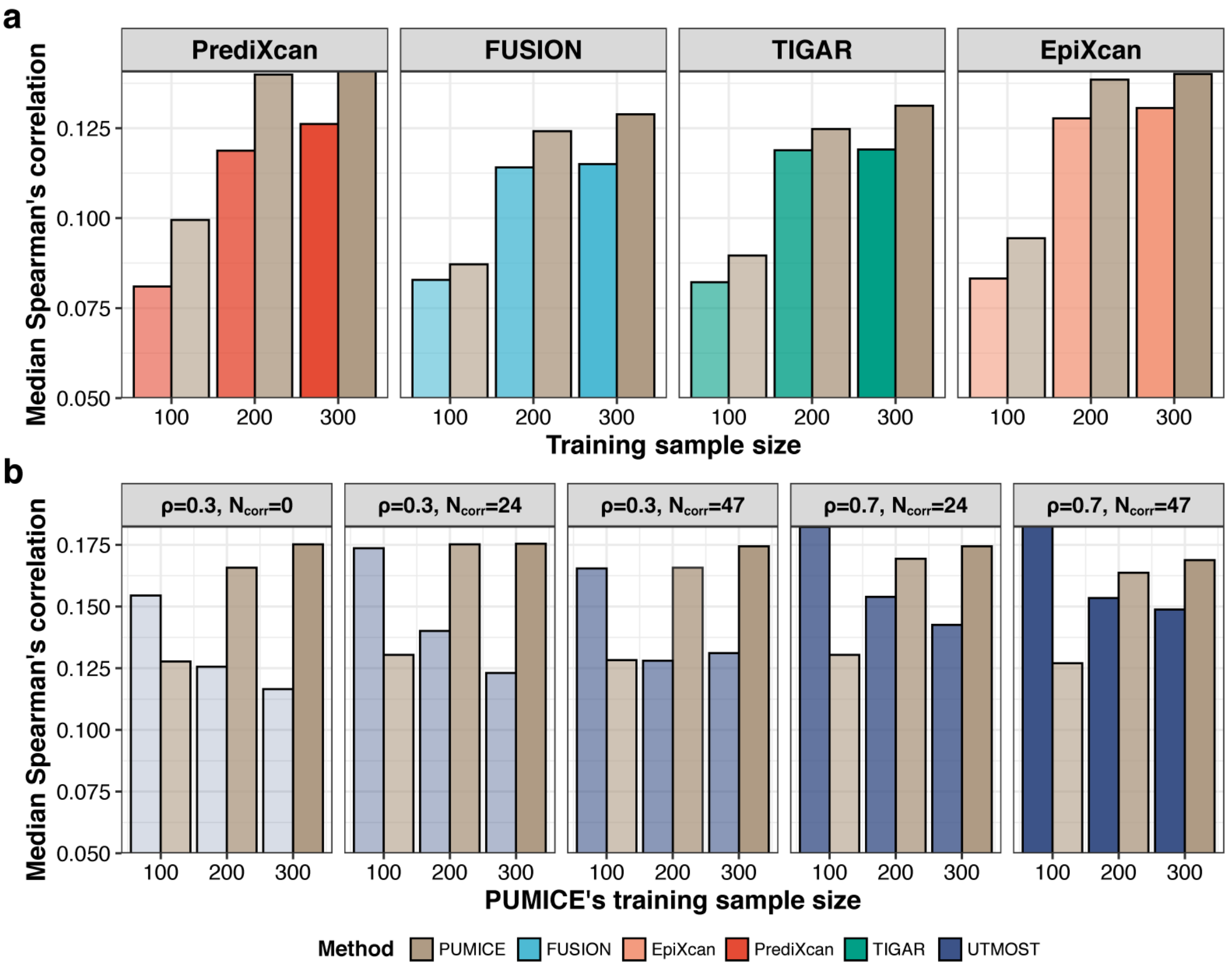

**Supplementary Figure 2: Quantile-quantile plots under null hypothesis.** Plots for single-tissue TWAS methods are illustrated for each training sample size: (**a**) 100, (**b**) 200, and (**c**) 300. Panel **d** shows a plot for multi-tissue TWAS method (i.e., UTMOST). Observed two-sided P-value associated with each gene is calculated according to the TWAS Z-score for gene-based association test. All methods have calibrated null distributions of p-values.

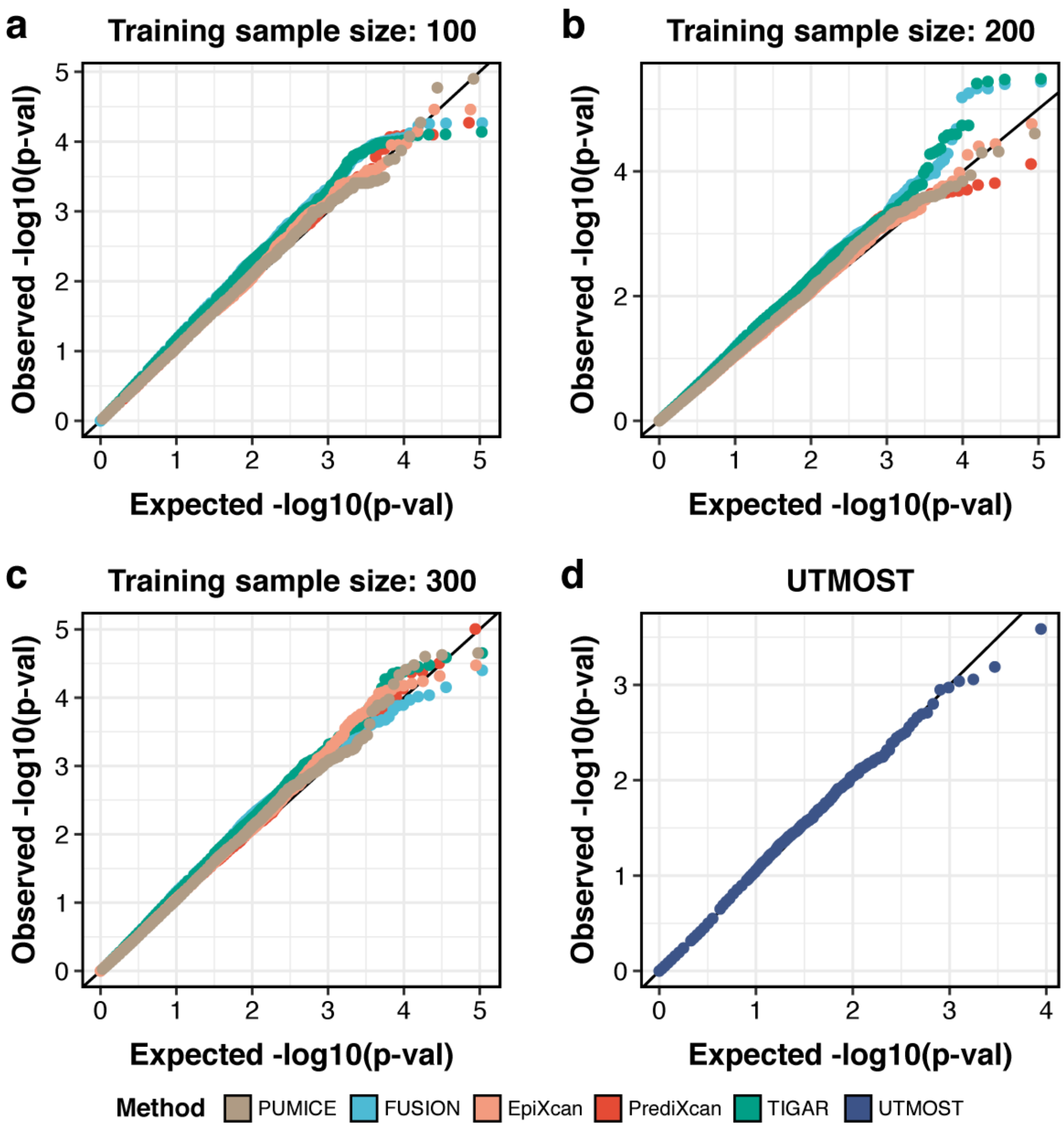

**Supplementary Figure 3: Comparison of prediction performance between PrediXcan and PUMICE across 48 tissues from GTEx V7.** Each panel represents a tissue. Spearman's rank correlation coefficient (between observed and predicted expression) is used as a metric to measure prediction performance. Only union of PrediXcan's and PUMICE's significant genes are included. White boxes represent the interquartile range (Q1-Q3). Whiskers represent the 1.5x interquartile range. Horizontal black lines represent the median values. Different colors represent different sample size ranges, with blue representing ≤150, red representing >150 and ≤250 and green representing >250. The sample size used to create each boxplot is provided in Supplementary Data 2.

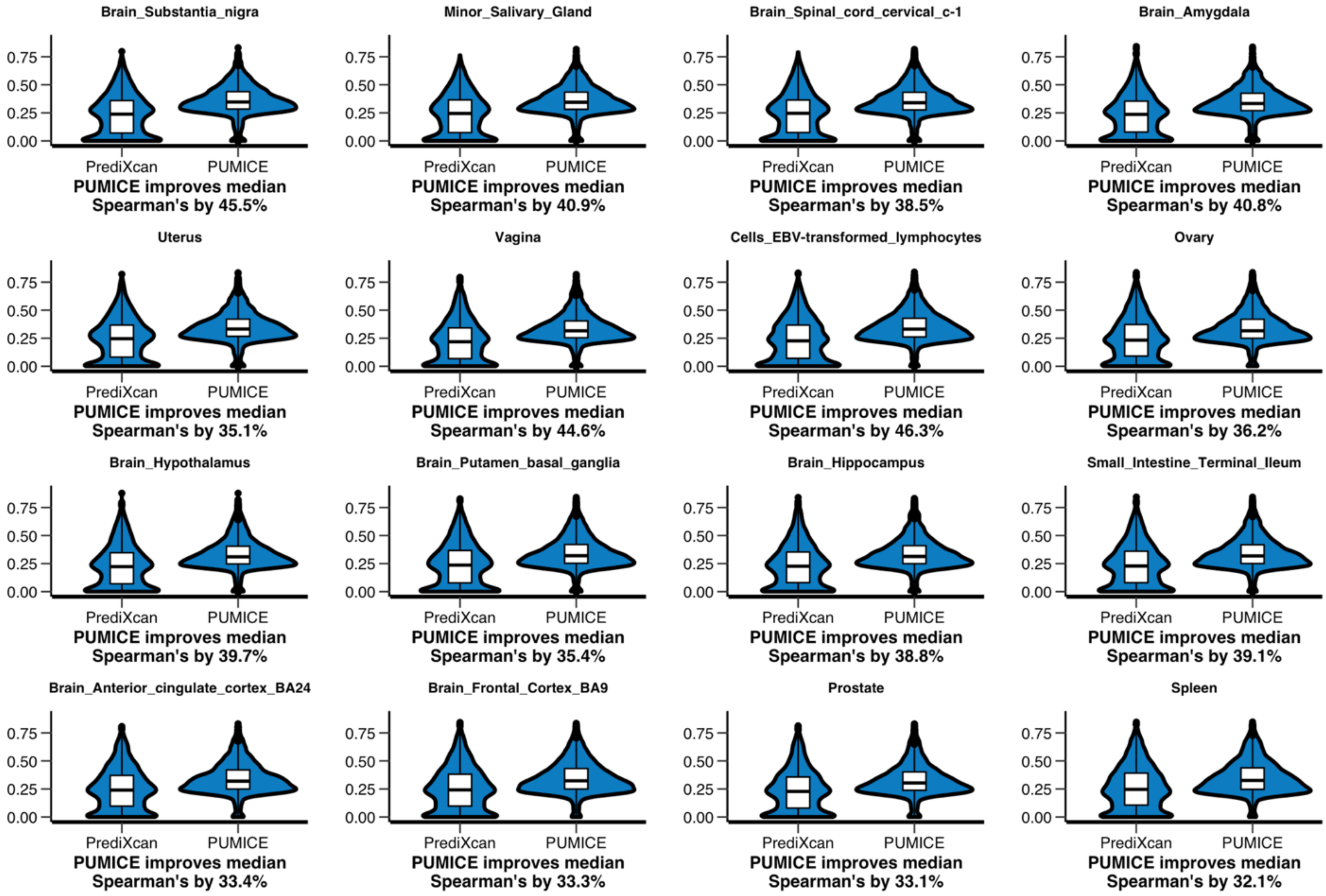

Brain_Substantia_nigra
Minor_Salivary_Gland
Brain_Spinal_cord_cervical_c-1
Brain_Amygdala
0.75
0.50
0.25
0.00
PrediXcan
PUMICE
PUMICE improves median Spearman's by 45.5%
PUMICE improves median Spearman's by 40.9%
PUMICE improves median Spearman's by 38.5%
PUMICE improves median Spearman's by 40.8%
Uterus
Vagina
Cells_EBV-transformed_lymphocytes
Ovary
PUMICE improves median Spearman's by 35.1%
PUMICE improves median Spearman's by 44.6%
PUMICE improves median Spearman's by 46.3%
PUMICE improves median Spearman's by 36.2%
Brain_Hypothalamus
Brain_Putamen_basal_ganglia
Brain_Hippocampus
Small_Intestine_Terminal_Ileum
PUMICE improves median Spearman's by 39.7%
PUMICE improves median Spearman's by 35.4%
PUMICE improves median Spearman's by 38.8%
PUMICE improves median Spearman's by 39.1%
Brain_Anterior_cingulate_cortex_BA24
Brain_Frontal_Cortex_BA9
Prostate
Spleen
PUMICE improves median Spearman's by 33.4%
PUMICE improves median Spearman's by 33.3%
PUMICE improves median Spearman's by 33.1%
PUMICE improves median Spearman's by 32.1%

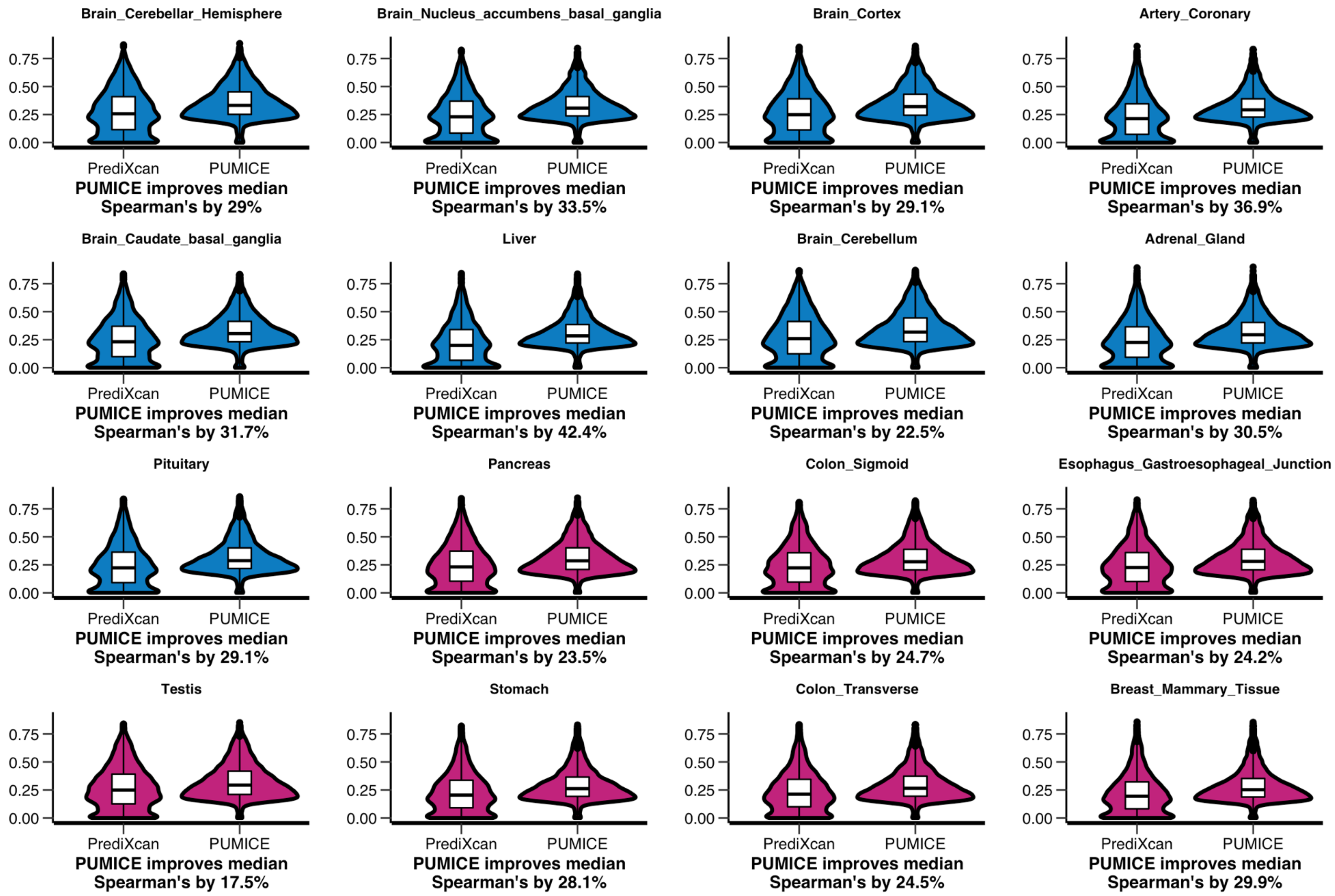
Brain_Cerebellar_Hemisphere
Brain_Nucleus_accumbens_basal_ganglia
Brain_Cortex
Artery_Coronary
Brain_Caudate_basal_ganglia
Liver
Brain_Cerebellum
Adrenal_Gland
Pituitary
Pancreas
Colon_Sigmoid
Esophagus_Gastroesophageal_Junction
Testis
Stomach
Colon_Transverse
Breast_Mammary_Tissue
0.75
0.50
0.25
0.00
PrediXcan
PUMICE
PUMICE improves median Spearman's by 29%
PUMICE improves median Spearman's by 33.5%
PUMICE improves median Spearman's by 29.1%
PUMICE improves median Spearman's by 36.9%
PUMICE improves median Spearman's by 31.7%
PUMICE improves median Spearman's by 42.4%
PUMICE improves median Spearman's by 22.5%
PUMICE improves median Spearman's by 30.5%
PUMICE improves median Spearman's by 29.1%
PUMICE improves median Spearman's by 23.5%
PUMICE improves median Spearman's by 24.7%
PUMICE improves median Spearman's by 24.2%
PUMICE improves median Spearman's by 17.5%
PUMICE improves median Spearman's by 28.1%
PUMICE improves median Spearman's by 24.5%
PUMICE improves median Spearman's by 29.9%

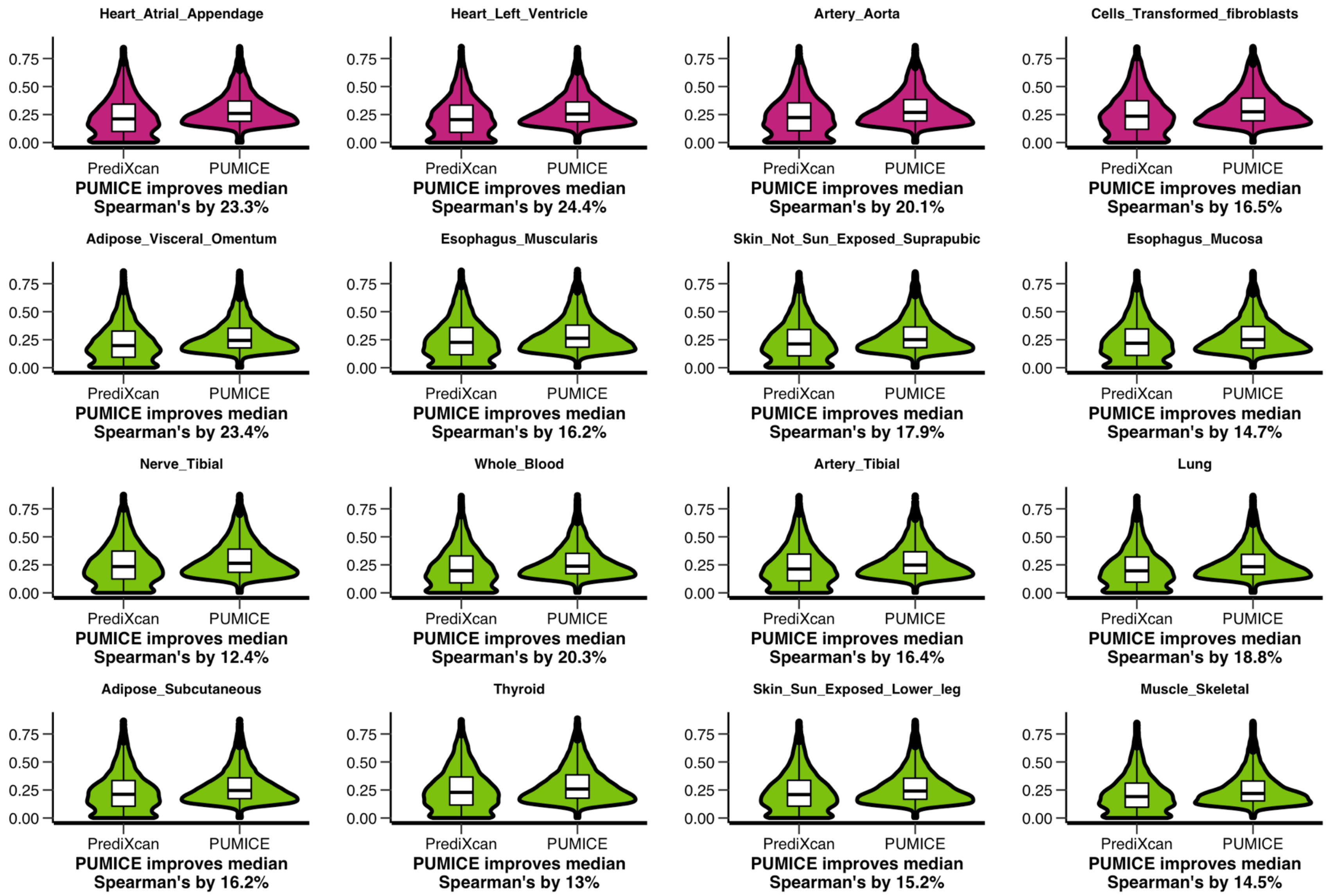
Heart_Atrial_Appendage
PUMICE improves median Spearman's by 23.3%
Heart_Left_Ventricle
PUMICE improves median Spearman's by 24.4%
Artery_Aorta
PUMICE improves median Spearman's by 20.1%
Cells_Transformed_fibroblasts
PUMICE improves median Spearman's by 16.5%
Adipose_Visceral_Omentum
PUMICE improves median Spearman's by 23.4%
Esophagus_Muscularis
PUMICE improves median Spearman's by 16.2%
Skin_Not_Sun_Exposed_Suprapubic
PUMICE improves median Spearman's by 17.9%
Esophagus_Mucosa
PUMICE improves median Spearman's by 14.7%
Nerve_Tibial
PUMICE improves median Spearman's by 12.4%
Whole_Blood
PUMICE improves median Spearman's by 20.3%
Artery_Tibial
PUMICE improves median Spearman's by 16.4%
Lung
PUMICE improves median Spearman's by 18.8%
Adipose_Subcutaneous
PUMICE improves median Spearman's by 16.2%
Thyroid
PUMICE improves median Spearman's by 13%
Skin_Sun_Exposed_Lower_leg
PUMICE improves median Spearman's by 15.2%
Muscle_Skeletal
PUMICE improves median Spearman's by 14.5%
PrediXcan
PUMICE
0.00
0.25
0.50
0.75

**Supplementary Figure 4: Comparison of prediction performance between FUSION and PUMICE across 48 tissues from GTEx V7.** Each panel represents a tissue. Spearman's rank correlation coefficient (between observed and predicted expression) is used as a metric to measure prediction performance. Only union of FUSION's and PUMICE's significant genes are included. White boxes represent the interquartile range (Q1-Q3). Whiskers represent the 1.5x interquartile range. Horizontal black lines represent the median values. Different colors represent different sample size ranges, with blue representing ≤150, red representing >150 and ≤250, and green representing >250. The sample size used to create each boxplot is provided in Supplementary Data 2.

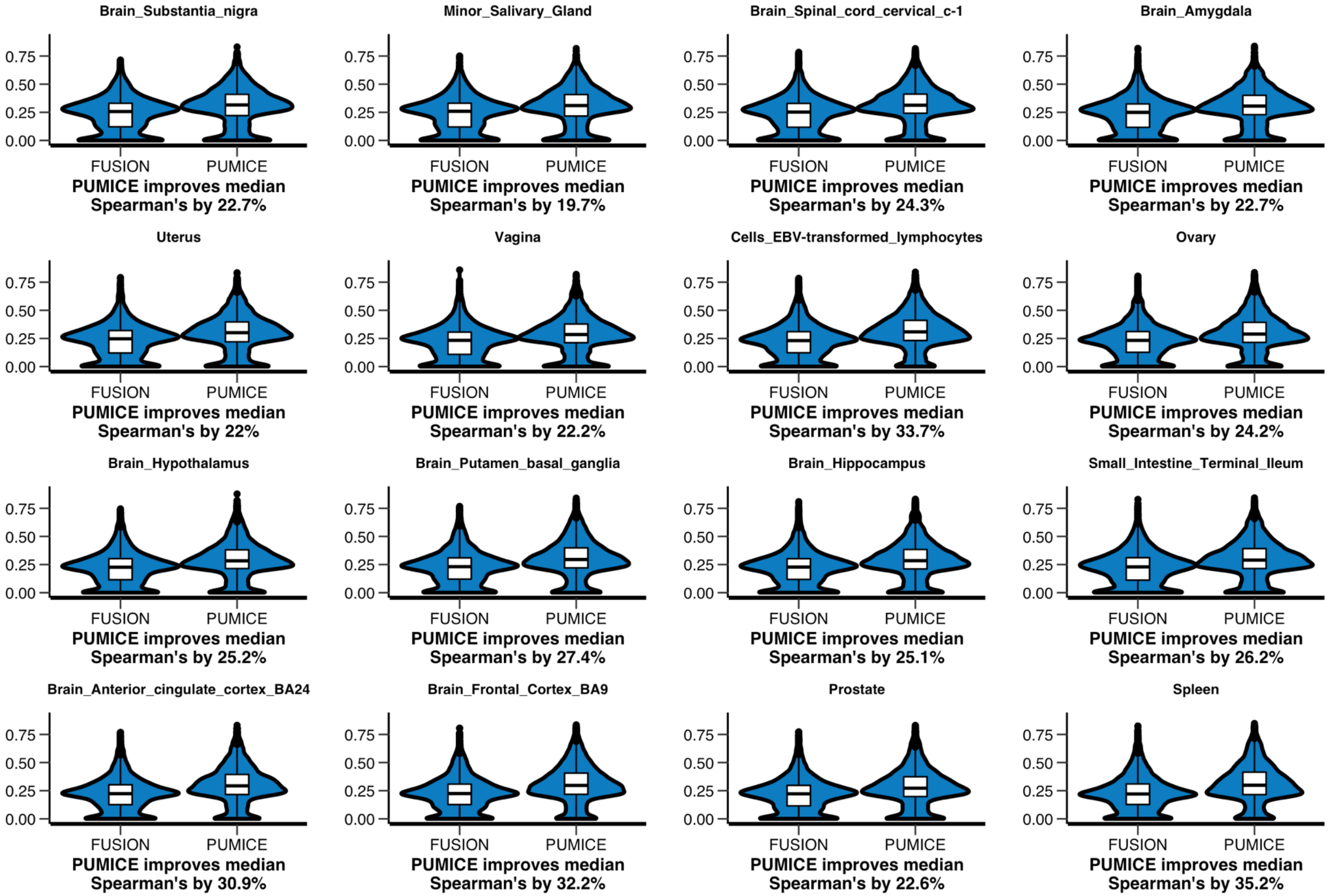
Brain_Substantia_nigra
0.75
0.50
0.25
0.00
FUSION
PUMICE
PUMICE improves median Spearman's by 22.7%
Minor_Salivary_Gland
FUSION
PUMICE
PUMICE improves median Spearman's by 19.7%
Brain_Spinal_cord_cervical_c-1
FUSION
PUMICE
PUMICE improves median Spearman's by 24.3%
Brain_Amygdala
FUSION
PUMICE
PUMICE improves median Spearman's by 22.7%
Uterus
FUSION
PUMICE
PUMICE improves median Spearman's by 22%
Vagina
FUSION
PUMICE
PUMICE improves median Spearman's by 22.2%
Cells_EBV-transformed_lymphocytes
FUSION
PUMICE
PUMICE improves median Spearman's by 33.7%
Ovary
FUSION
PUMICE
PUMICE improves median Spearman's by 24.2%
Brain_Hypothalamus
FUSION
PUMICE
PUMICE improves median Spearman's by 25.2%
Brain_Putamen_basal_ganglia
FUSION
PUMICE
PUMICE improves median Spearman's by 27.4%
Brain_Hippocampus
FUSION
PUMICE
PUMICE improves median Spearman's by 25.1%
Small_Intestine_Terminal_Ileum
FUSION
PUMICE
PUMICE improves median Spearman's by 26.2%
Brain_Anterior_cingulate_cortex_BA24
FUSION
PUMICE
PUMICE improves median Spearman's by 30.9%
Brain_Frontal_Cortex_BA9
FUSION
PUMICE
PUMICE improves median Spearman's by 32.2%
Prostate
FUSION
PUMICE
PUMICE improves median Spearman's by 22.6%
Spleen
FUSION
PUMICE
PUMICE improves median Spearman's by 35.2%

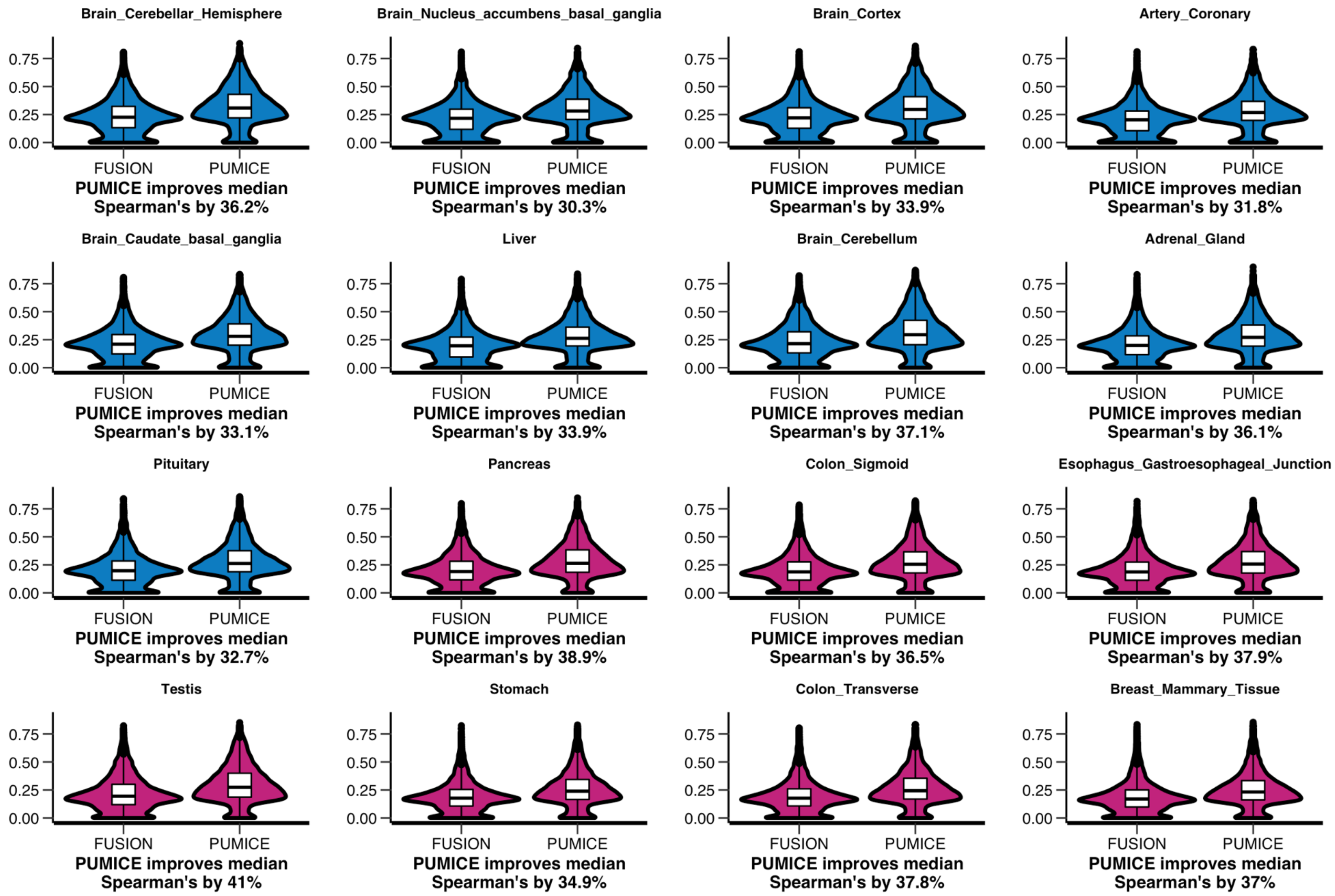
Brain_Cerebellar_Hemisphere
0.75
0.50
0.25
0.00
FUSION
PUMICE
PUMICE improves median
Spearman's by 36.2%
Brain_Nucleus_accumbens_basal_ganglia
FUSION
PUMICE
PUMICE improves median
Spearman's by 30.3%
Brain_Cortex
FUSION
PUMICE
PUMICE improves median
Spearman's by 33.9%
Artery_Coronary
FUSION
PUMICE
PUMICE improves median
Spearman's by 31.8%
Brain_Caudate_basal_ganglia
FUSION
PUMICE
PUMICE improves median
Spearman's by 33.1%
Liver
FUSION
PUMICE
PUMICE improves median
Spearman's by 33.9%
Brain_Cerebellum
FUSION
PUMICE
PUMICE improves median
Spearman's by 37.1%
Adrenal_Gland
FUSION
PUMICE
PUMICE improves median
Spearman's by 36.1%
Pituitary
FUSION
PUMICE
PUMICE improves median
Spearman's by 32.7%
Pancreas
FUSION
PUMICE
PUMICE improves median
Spearman's by 38.9%
Colon_Sigmoid
FUSION
PUMICE
PUMICE improves median
Spearman's by 36.5%
Esophagus_Gastroesophageal_Junction
FUSION
PUMICE
PUMICE improves median
Spearman's by 37.9%
Testis
FUSION
PUMICE
PUMICE improves median
Spearman's by 41%
Stomach
FUSION
PUMICE
PUMICE improves median
Spearman's by 34.9%
Colon_Transverse
FUSION
PUMICE
PUMICE improves median
Spearman's by 37.8%
Breast_Mammary_Tissue
FUSION
PUMICE
PUMICE improves median
Spearman's by 37%

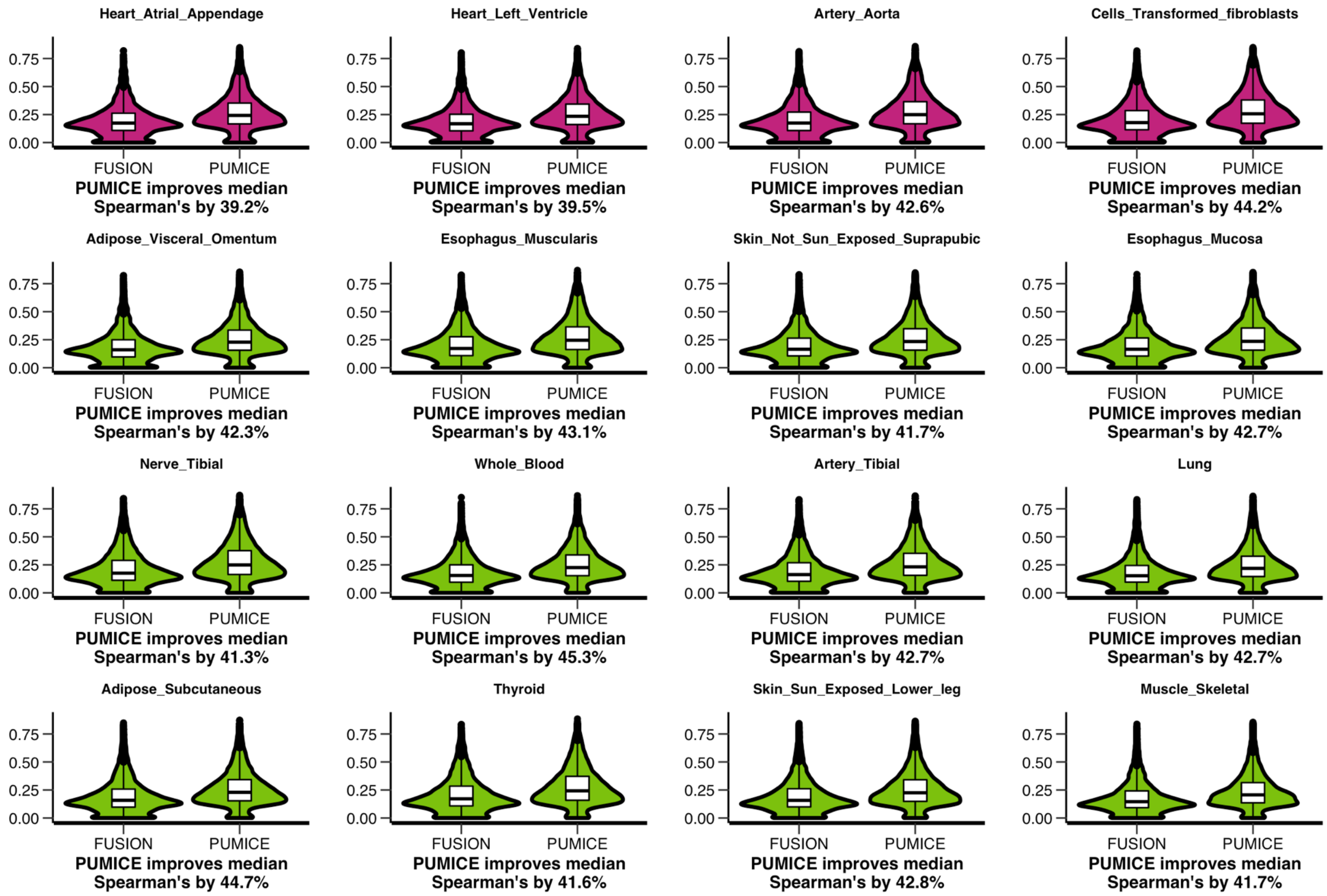
Heart_Atrial_Appendage
0.75
0.50
0.25
0.00
FUSION
PUMICE
PUMICE improves median
Spearman's by 39.2%
Heart_Left_Ventricle
PUMICE improves median
Spearman's by 39.5%
Artery_Aorta
PUMICE improves median
Spearman's by 42.6%
Cells_Transformed_fibroblasts
PUMICE improves median
Spearman's by 44.2%
Adipose_Visceral_Omentum
PUMICE improves median
Spearman's by 42.3%
Esophagus_Muscularis
PUMICE improves median
Spearman's by 43.1%
Skin_Not_Sun_Exposed_Suprapubic
PUMICE improves median
Spearman's by 41.7%
Esophagus_Mucosa
PUMICE improves median
Spearman's by 42.7%
Nerve_Tibial
PUMICE improves median
Spearman's by 41.3%
Whole_Blood
PUMICE improves median
Spearman's by 45.3%
Artery_Tibial
PUMICE improves median
Spearman's by 42.7%
Lung
PUMICE improves median
Spearman's by 42.7%
Adipose_Subcutaneous
PUMICE improves median
Spearman's by 44.7%
Thyroid
PUMICE improves median
Spearman's by 41.6%
Skin_Sun_Exposed_Lower_leg
PUMICE improves median
Spearman's by 42.8%
Muscle_Skeletal
PUMICE improves median
Spearman's by 41.7%

**Supplementary Figure 5: Comparison of prediction performance between TIGAR and PUMICE across 48 tissues from GTEx V7.** Each panel represents a tissue. Spearman's rank correlation coefficient (between observed and predicted expression) is used as a metric to measure prediction performance. Only union of TIGAR's and PUMICE's significant genes are included. White boxes represent the interquartile range (Q1-Q3). Whiskers represent the 1.5x interquartile range. Horizontal black lines represent the median values. Different colors represent different sample size ranges, with blue representing ≤150, red representing >150 and ≤250, and green representing >250. The sample size used to create each boxplot is provided in Supplementary Data 2.

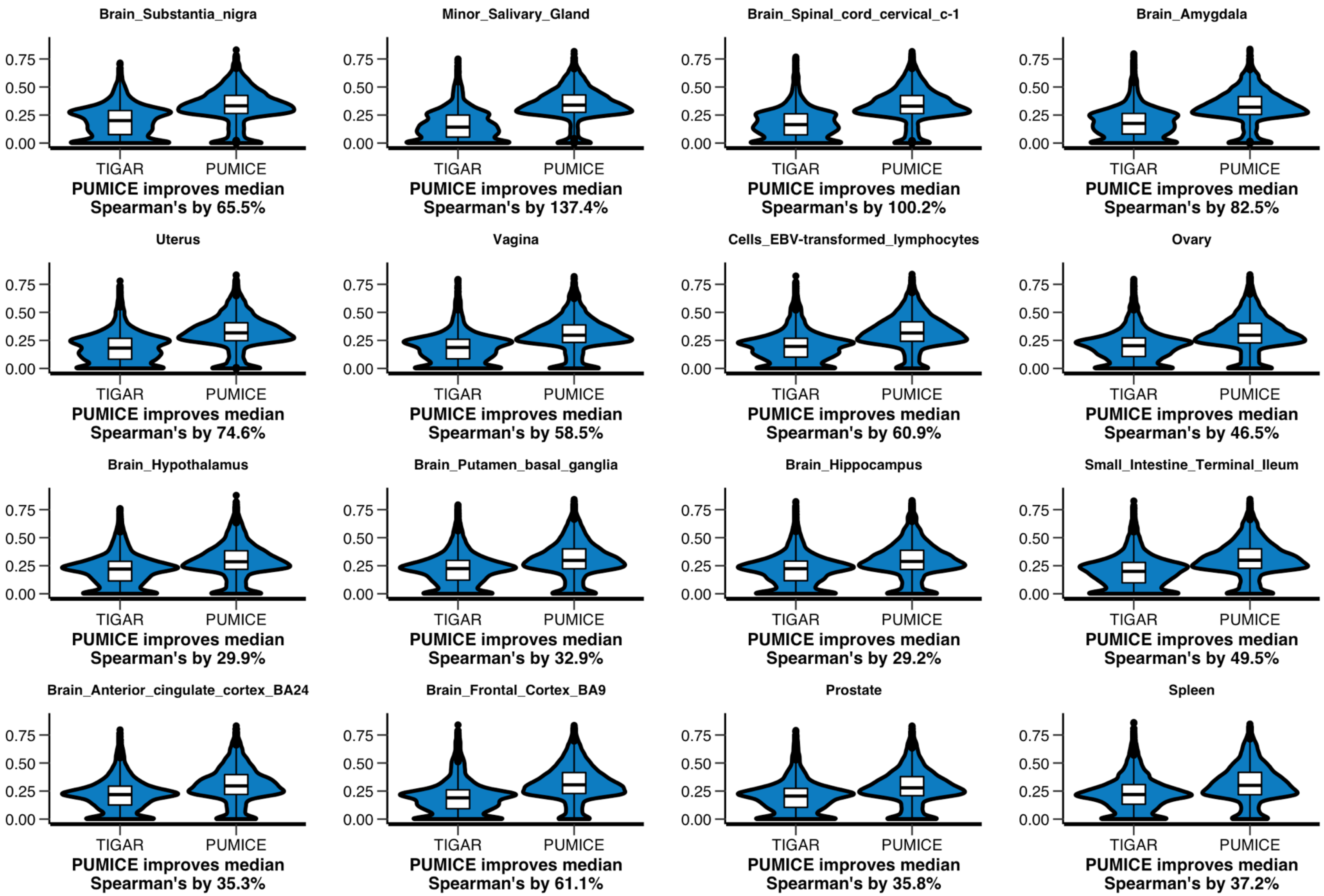
Brain_Substantia_nigra
PUMICE improves median Spearman's by 65.5%
Minor_Salivary_Gland
PUMICE improves median Spearman's by 137.4%
Brain_Spinal_cord_cervical_c-1
PUMICE improves median Spearman's by 100.2%
Brain_Amygdala
PUMICE improves median Spearman's by 82.5%
Uterus
PUMICE improves median Spearman's by 74.6%
Vagina
PUMICE improves median Spearman's by 58.5%
Cells_EBV-transformed_lymphocytes
PUMICE improves median Spearman's by 60.9%
Ovary
PUMICE improves median Spearman's by 46.5%
Brain_Hypothalamus
PUMICE improves median Spearman's by 29.9%
Brain_Putamen_basal_ganglia
PUMICE improves median Spearman's by 32.9%
Brain_Hippocampus
PUMICE improves median Spearman's by 29.2%
Small_Intestine_Terminal_Ileum
PUMICE improves median Spearman's by 49.5%
Brain_Anterior_cingulate_cortex_BA24
PUMICE improves median Spearman's by 35.3%
Brain_Frontal_Cortex_BA9
PUMICE improves median Spearman's by 61.1%
Prostate
PUMICE improves median Spearman's by 35.8%
Spleen
PUMICE improves median Spearman's by 37.2%
TIGAR
PUMICE
0.00
0.25
0.50
0.75

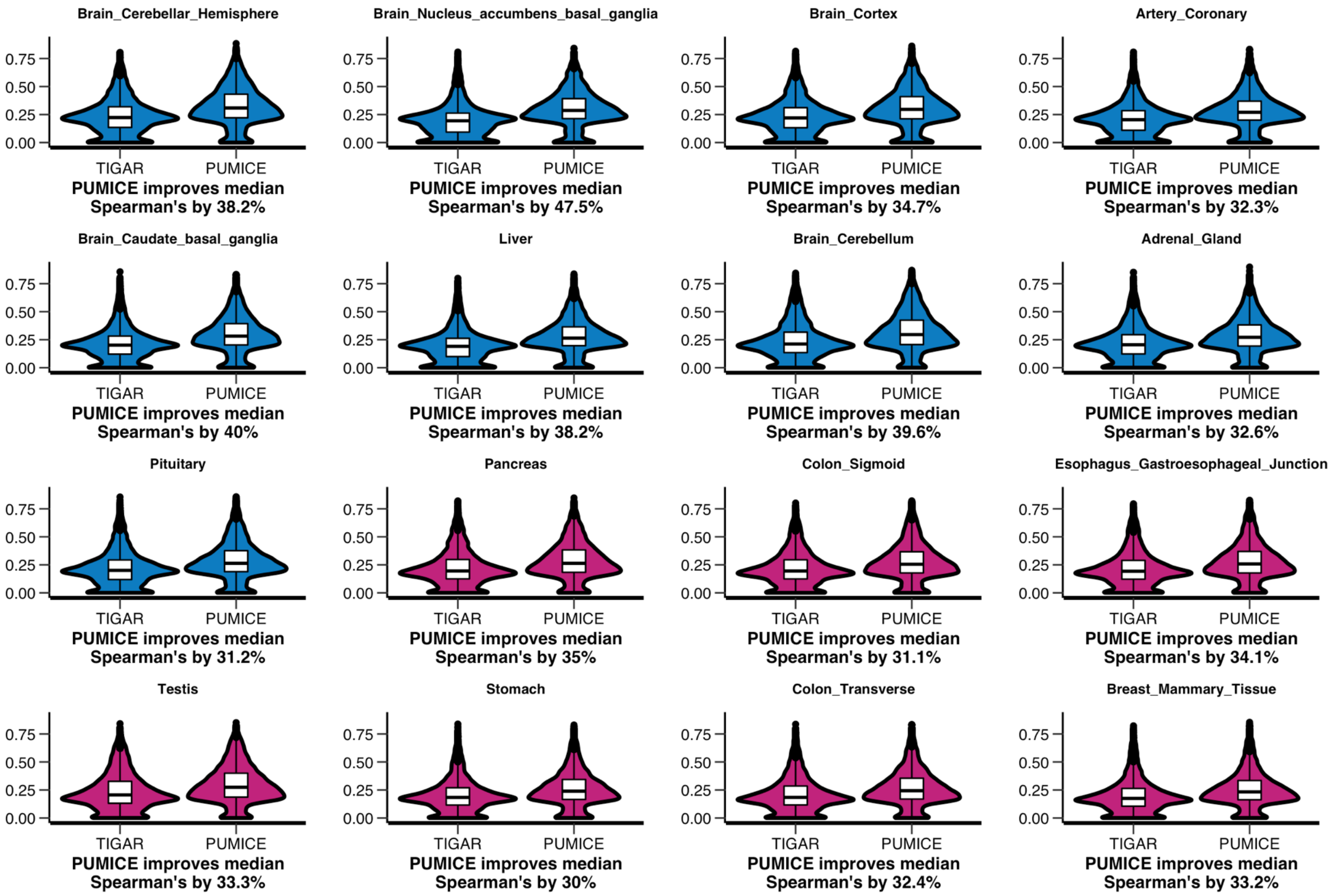
Brain_Cerebellar_Hemisphere
Brain_Nucleus_accumbens_basal_ganglia
Brain_Cortex
Artery_Coronary
0.75
0.50
0.25
0.00
TIGAR
PUMICE
PUMICE improves median Spearman's by 38.2%
PUMICE improves median Spearman's by 47.5%
PUMICE improves median Spearman's by 34.7%
PUMICE improves median Spearman's by 32.3%
Brain_Caudate_basal_ganglia
Liver
Brain_Cerebellum
Adrenal_Gland
PUMICE improves median Spearman's by 40%
PUMICE improves median Spearman's by 38.2%
PUMICE improves median Spearman's by 39.6%
PUMICE improves median Spearman's by 32.6%
Pituitary
Pancreas
Colon_Sigmoid
Esophagus_Gastroesophageal_Junction
PUMICE improves median Spearman's by 31.2%
PUMICE improves median Spearman's by 35%
PUMICE improves median Spearman's by 31.1%
PUMICE improves median Spearman's by 34.1%
Testis
Stomach
Colon_Transverse
Breast_Mammary_Tissue
PUMICE improves median Spearman's by 33.3%
PUMICE improves median Spearman's by 30%
PUMICE improves median Spearman's by 32.4%
PUMICE improves median Spearman's by 33.2%

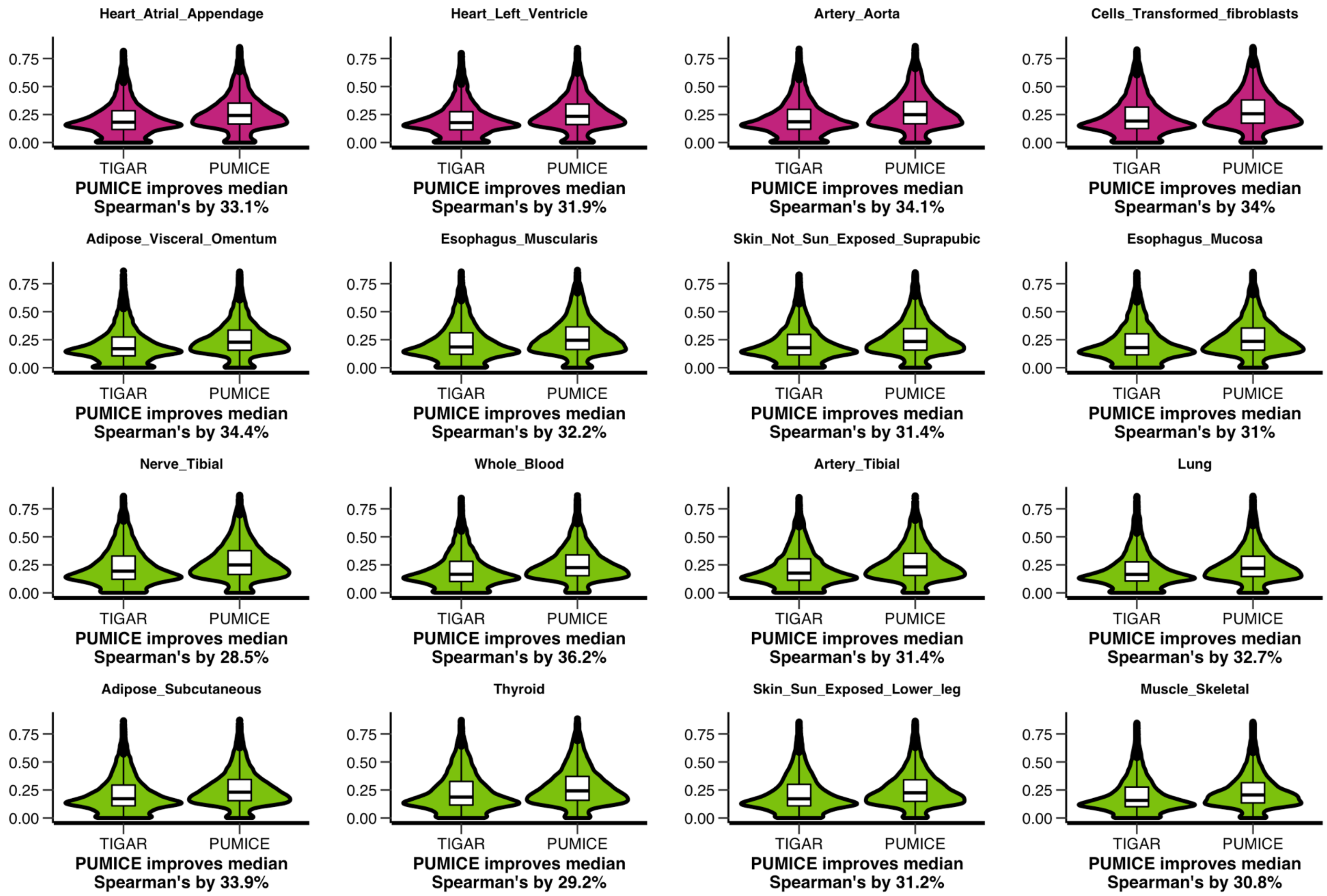

Heart_Atrial_Appendage
0.75
0.50
0.25
0.00
TIGAR
PUMICE
PUMICE improves median
Spearman's by 33.1%
Heart_Left_Ventricle
PUMICE improves median
Spearman's by 31.9%
Artery_Aorta
PUMICE improves median
Spearman's by 34.1%
Cells_Transformed_fibroblasts
PUMICE improves median
Spearman's by 34%
Adipose_Visceral_Omentum
PUMICE improves median
Spearman's by 34.4%
Esophagus_Muscularis
PUMICE improves median
Spearman's by 32.2%
Skin_Not_Sun_Exposed_Suprapubic
PUMICE improves median
Spearman's by 31.4%
Esophagus_Mucosa
PUMICE improves median
Spearman's by 31%
Nerve_Tibial
PUMICE improves median
Spearman's by 28.5%
Whole_Blood
PUMICE improves median
Spearman's by 36.2%
Artery_Tibial
PUMICE improves median
Spearman's by 31.4%
Lung
PUMICE improves median
Spearman's by 32.7%
Adipose_Subcutaneous
PUMICE improves median
Spearman's by 33.9%
Thyroid
PUMICE improves median
Spearman's by 29.2%
Skin_Sun_Exposed_Lower_leg
PUMICE improves median
Spearman's by 31.2%
Muscle_Skeletal
PUMICE improves median
Spearman's by 30.8%

**Supplementary Figure 6: Comparison of prediction performance between EpiXcan and PUMICE across 8 tissues from GTEx V7.** Each panel represents a tissue for which an EpiXcan model was computed. Spearman's rank correlation coefficient (between observed and predicted expression) is used as a metric to measure prediction performance. Only union of EpiXcan's and PUMICE's significant genes are included. White boxes represent the interquartile range (Q1-Q3). Whiskers represent the 1.5x interquartile range. Horizontal black lines represent the median values. Different colors represent different sample size ranges, with blue representing ≤150, red representing >150 and ≤250, and green representing >250. The sample size used to create each boxplot is provided in Supplementary Data 2.

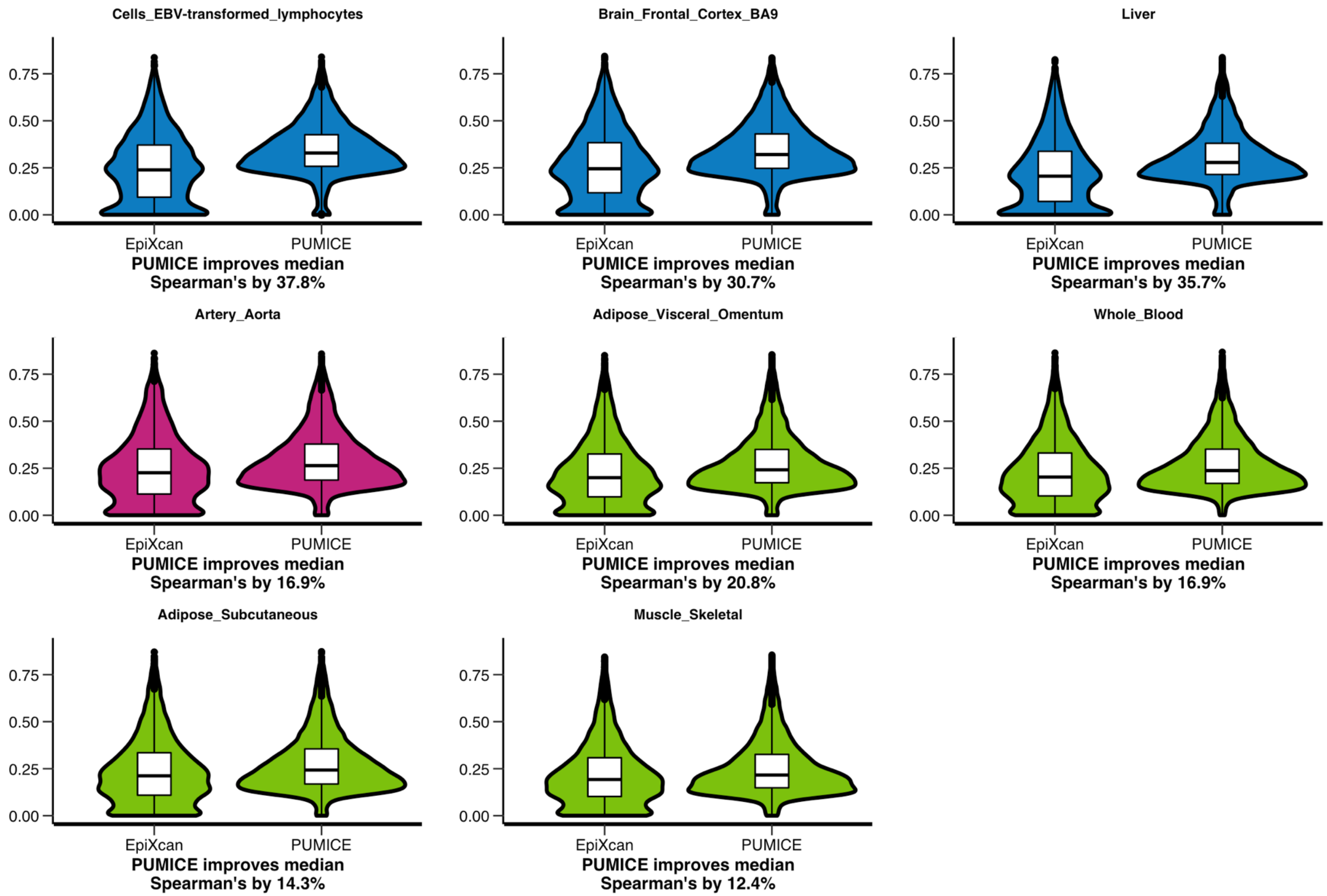
Cells_EBV-transformed_lymphocytes
0.75
0.50
0.25
0.00
EpiXcan
PUMICE
PUMICE improves median Spearman's by 37.8%
Brain_Frontal_Cortex_BA9
EpiXcan
PUMICE
PUMICE improves median Spearman's by 30.7%
Liver
EpiXcan
PUMICE
PUMICE improves median Spearman's by 35.7%
Artery_Aorta
EpiXcan
PUMICE
PUMICE improves median Spearman's by 16.9%
Adipose_Visceral_Omentum
EpiXcan
PUMICE
PUMICE improves median Spearman's by 20.8%
Whole_Blood
EpiXcan
PUMICE
PUMICE improves median Spearman's by 16.9%
Adipose_Subcutaneous
EpiXcan
PUMICE
PUMICE improves median Spearman's by 14.3%
Muscle_Skeletal
EpiXcan
PUMICE
PUMICE improves median Spearman's by 12.4%

**Supplementary Figure 7: Comparison of prediction performance between UTMOST and PUMICE across 48 tissues from GTEx V7.** Each panel represents a tissue. Spearman's rank correlation coefficient (between observed and predicted expression) is used as a metric to measure prediction performance. Only union of UTMOST's and PUMICE's significant genes are included. White boxes represent the interquartile range (Q1-Q3). Whiskers represent the 1.5x interquartile range. Horizontal black lines represent the median values. Different colors represent different sample size ranges, with blue representing ≤150, red representing >150 and ≤250, and green representing >250. The sample size used to create each boxplot is provided in Supplementary Data 2.

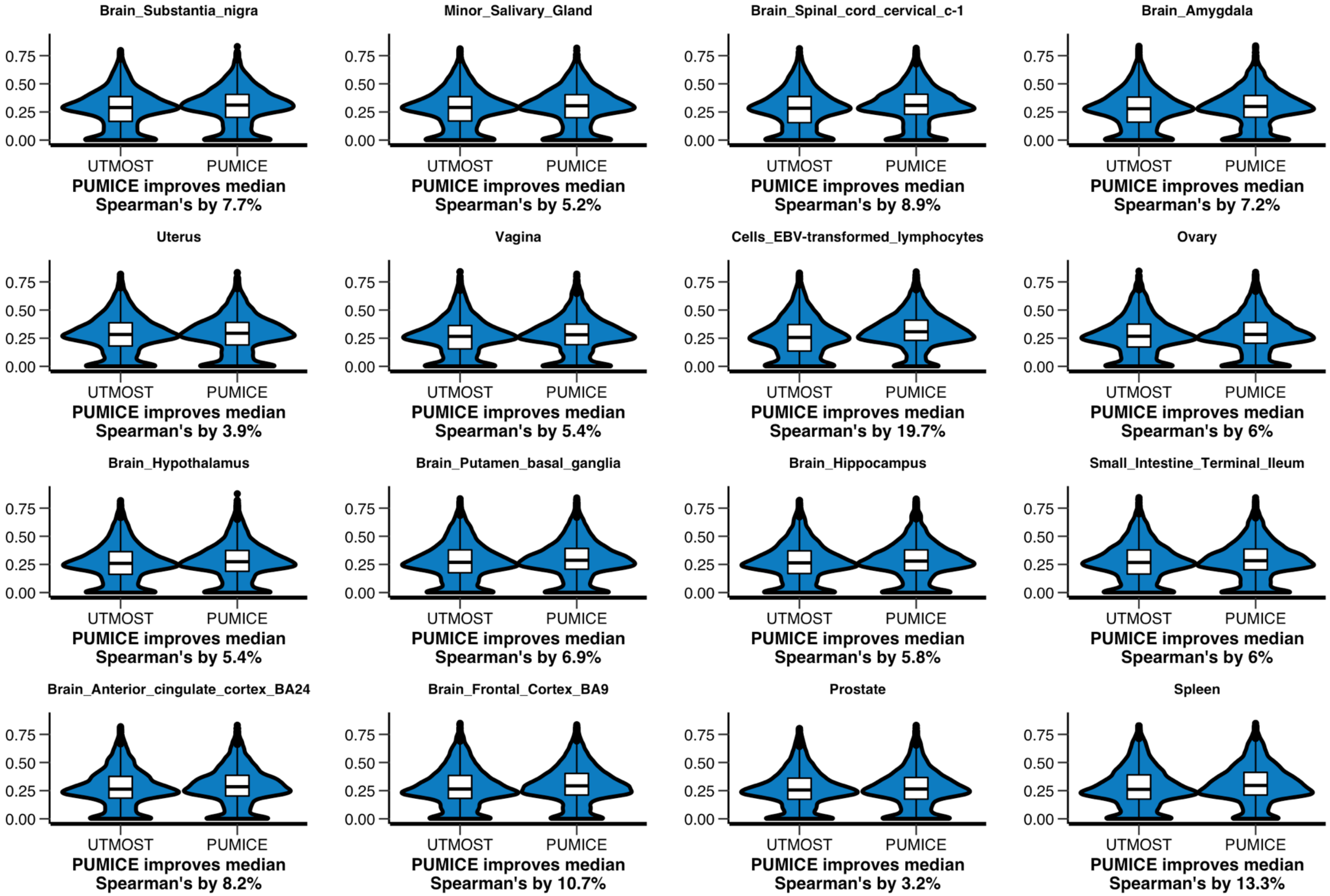
Brain_Substantia_nigra
0.75
0.50
0.25
0.00
UTMOST
PUMICE
PUMICE improves median
Spearman's by 7.7%
Minor_Salivary_Gland
UTMOST
PUMICE
PUMICE improves median
Spearman's by 5.2%
Brain_Spinal_cord_cervical_c-1
UTMOST
PUMICE
PUMICE improves median
Spearman's by 8.9%
Brain_Amygdala
UTMOST
PUMICE
PUMICE improves median
Spearman's by 7.2%
Uterus
UTMOST
PUMICE
PUMICE improves median
Spearman's by 3.9%
Vagina
UTMOST
PUMICE
PUMICE improves median
Spearman's by 5.4%
Cells_EBV-transformed_lymphocytes
UTMOST
PUMICE
PUMICE improves median
Spearman's by 19.7%
Ovary
UTMOST
PUMICE
PUMICE improves median
Spearman's by 6%
Brain_Hypothalamus
UTMOST
PUMICE
PUMICE improves median
Spearman's by 5.4%
Brain_Putamen_basal_ganglia
UTMOST
PUMICE
PUMICE improves median
Spearman's by 6.9%
Brain_Hippocampus
UTMOST
PUMICE
PUMICE improves median
Spearman's by 5.8%
Small_Intestine_Terminal_Ileum
UTMOST
PUMICE
PUMICE improves median
Spearman's by 6%
Brain_Anterior_cingulate_cortex_BA24
UTMOST
PUMICE
PUMICE improves median
Spearman's by 8.2%
Brain_Frontal_Cortex_BA9
UTMOST
PUMICE
PUMICE improves median
Spearman's by 10.7%
Prostate
UTMOST
PUMICE
PUMICE improves median
Spearman's by 3.2%
Spleen
UTMOST
PUMICE
PUMICE improves median
Spearman's by 13.3%

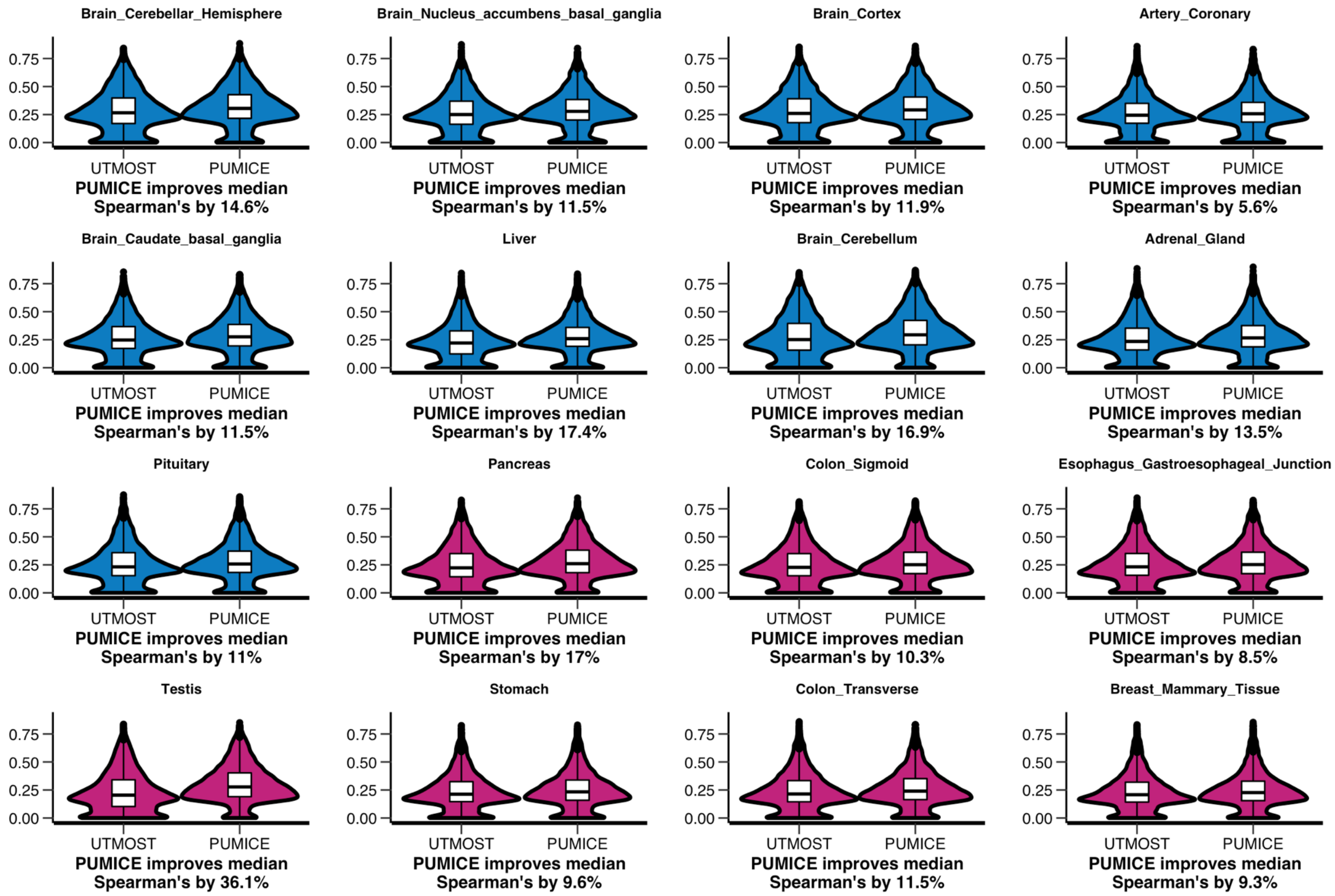
Brain_Cerebellar_Hemisphere
0.75
0.50
0.25
0.00
UTMOST
PUMICE
PUMICE improves median Spearman's by 14.6%
Brain_Nucleus_accumbens_basal_ganglia
PUMICE improves median Spearman's by 11.5%
Brain_Cortex
PUMICE improves median Spearman's by 11.9%
Artery_Coronary
PUMICE improves median Spearman's by 5.6%
Brain_Caudate_basal_ganglia
PUMICE improves median Spearman's by 11.5%
Liver
PUMICE improves median Spearman's by 17.4%
Brain_Cerebellum
PUMICE improves median Spearman's by 16.9%
Adrenal_Gland
PUMICE improves median Spearman's by 13.5%
Pituitary
PUMICE improves median Spearman's by 11%
Pancreas
PUMICE improves median Spearman's by 17%
Colon_Sigmoid
PUMICE improves median Spearman's by 10.3%
Esophagus_Gastroesophageal_Junction
PUMICE improves median Spearman's by 8.5%
Testis
PUMICE improves median Spearman's by 36.1%
Stomach
PUMICE improves median Spearman's by 9.6%
Colon_Transverse
PUMICE improves median Spearman's by 11.5%
Breast_Mammary_Tissue
PUMICE improves median Spearman's by 9.3%

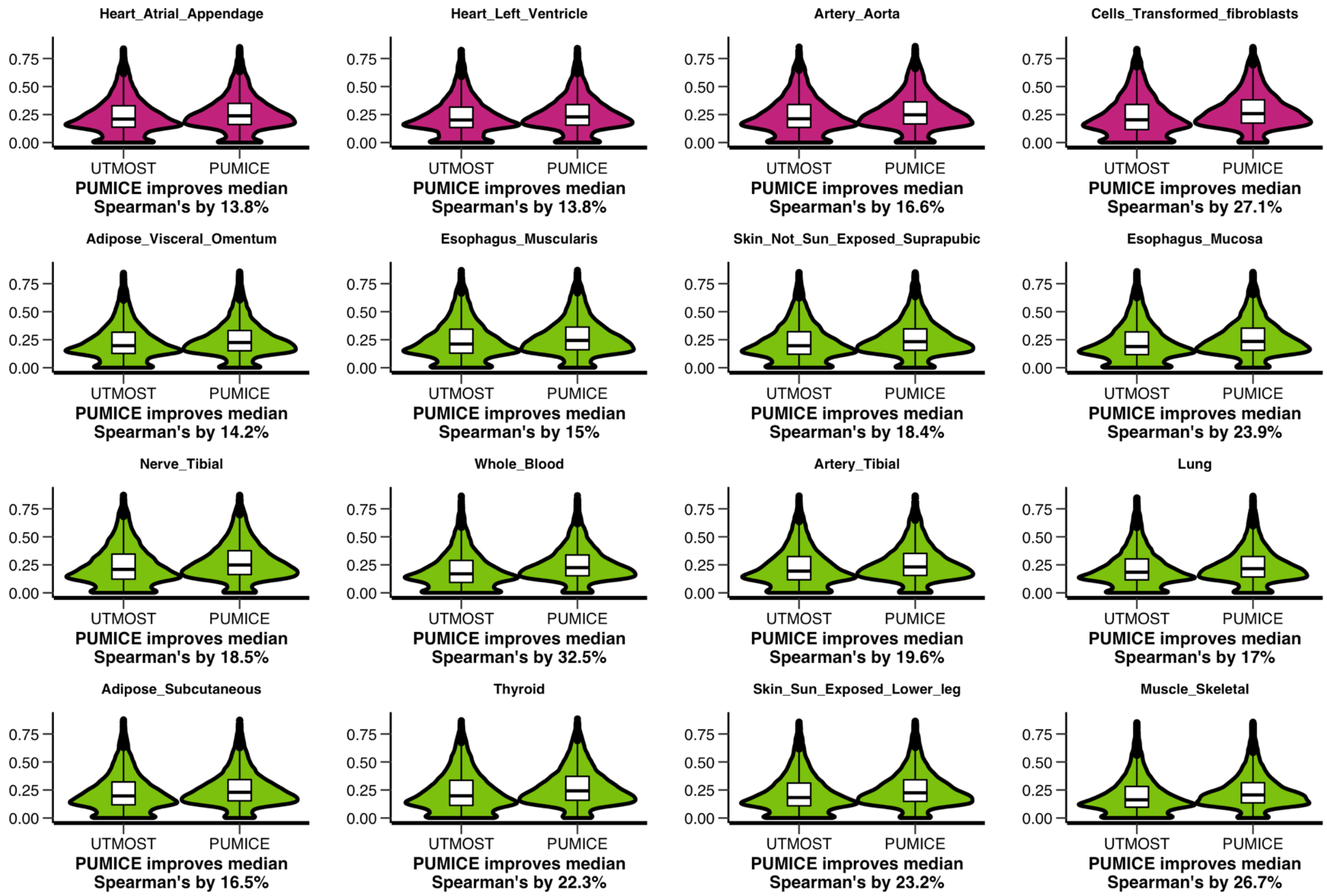
Heart_Atrial_Appendage
PUMICE improves median Spearman's by 13.8%
Heart_Left_Ventricle
PUMICE improves median Spearman's by 13.8%
Artery_Aorta
PUMICE improves median Spearman's by 16.6%
Cells_Transformed_fibroblasts
PUMICE improves median Spearman's by 27.1%
Adipose_Visceral_Omentum
PUMICE improves median Spearman's by 14.2%
Esophagus_Muscularis
PUMICE improves median Spearman's by 15%
Skin_Not_Sun_Exposed_Suprapubic
PUMICE improves median Spearman's by 18.4%
Esophagus_Mucosa
PUMICE improves median Spearman's by 23.9%
Nerve_Tibial
PUMICE improves median Spearman's by 18.5%
Whole_Blood
PUMICE improves median Spearman's by 32.5%
Artery_Tibial
PUMICE improves median Spearman's by 19.6%
Lung
PUMICE improves median Spearman's by 17%
Adipose_Subcutaneous
PUMICE improves median Spearman's by 16.5%
Thyroid
PUMICE improves median Spearman's by 22.3%
Skin_Sun_Exposed_Lower_leg
PUMICE improves median Spearman's by 23.2%
Muscle_Skeletal
PUMICE improves median Spearman's by 26.7%
UTMOST
PUMICE
0.00
0.25
0.50
0.75

**Supplementary Figure 8:** Comparison of median Spearman's rank correlation coefficient in real external datasets between PUMICE and other single-tissue TWAS methods. We plot the percent gains of Spearman’s correlation of PUMICE over other methods across three different external test datasets.

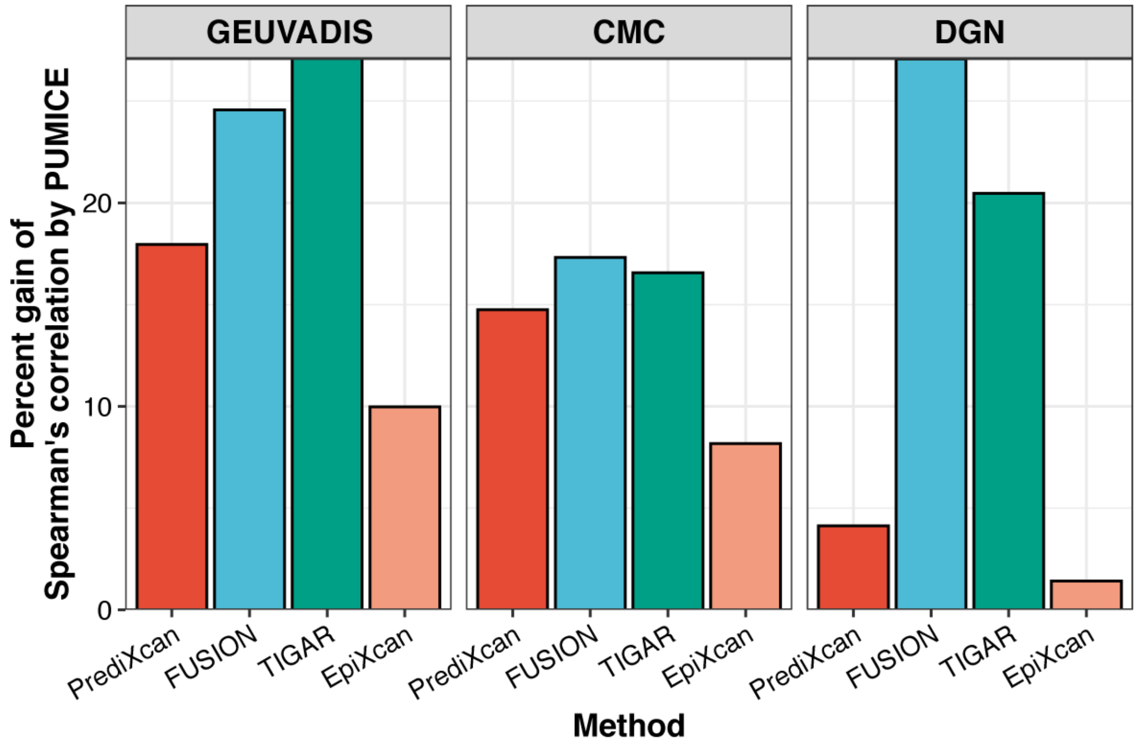

**Supplementary Figure 9: Quantile-quantile plots of p-values from different TWAS methods for all traits and COVID19 traits.** (**a**) QQ plots for PrediXcan, FUSION, TIGAR, PUMICE, and UTMOST across all traits. (**b**) QQ plots for PrediXcan+, FUSION+, TIGAR+, and PUMICE+ across all traits. (**c**) QQ plots for PrediXcan, FUSION, TIGAR, PUMICE, and UTMOST for COVID traits. (**d**) QQ plots for PrediXcan+, FUSION+, TIGAR+, and PUMICE+ for COVID traits. Observed two-sided P-value associated with each gene is calculated according to the TWAS Z-score for gene-based association test.

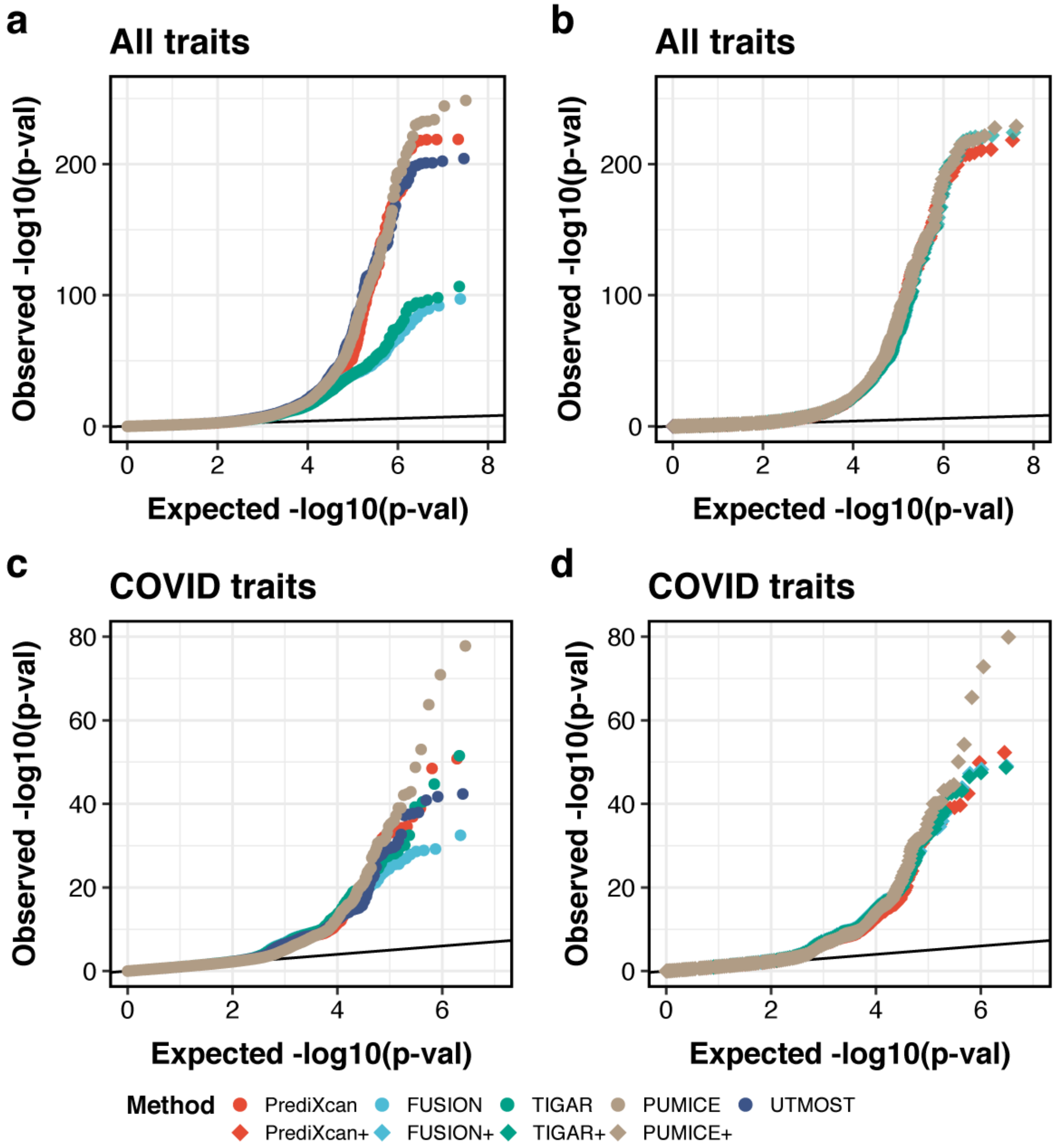

**Supplementary Figure 10. GTEx tissues structure plot.** Structure plot of estimated membership proportions using Grade of Membership model with K = 20 clusters. Model is fitted using 8,547 samples from 48 tissues in GTEx data. Each horizontal bar represents the cluster membership proportions for a single sample. Brain tissues are not shown in this plot, but are separately shown in Supplementary Figure 11 to highlight refined brain structures.

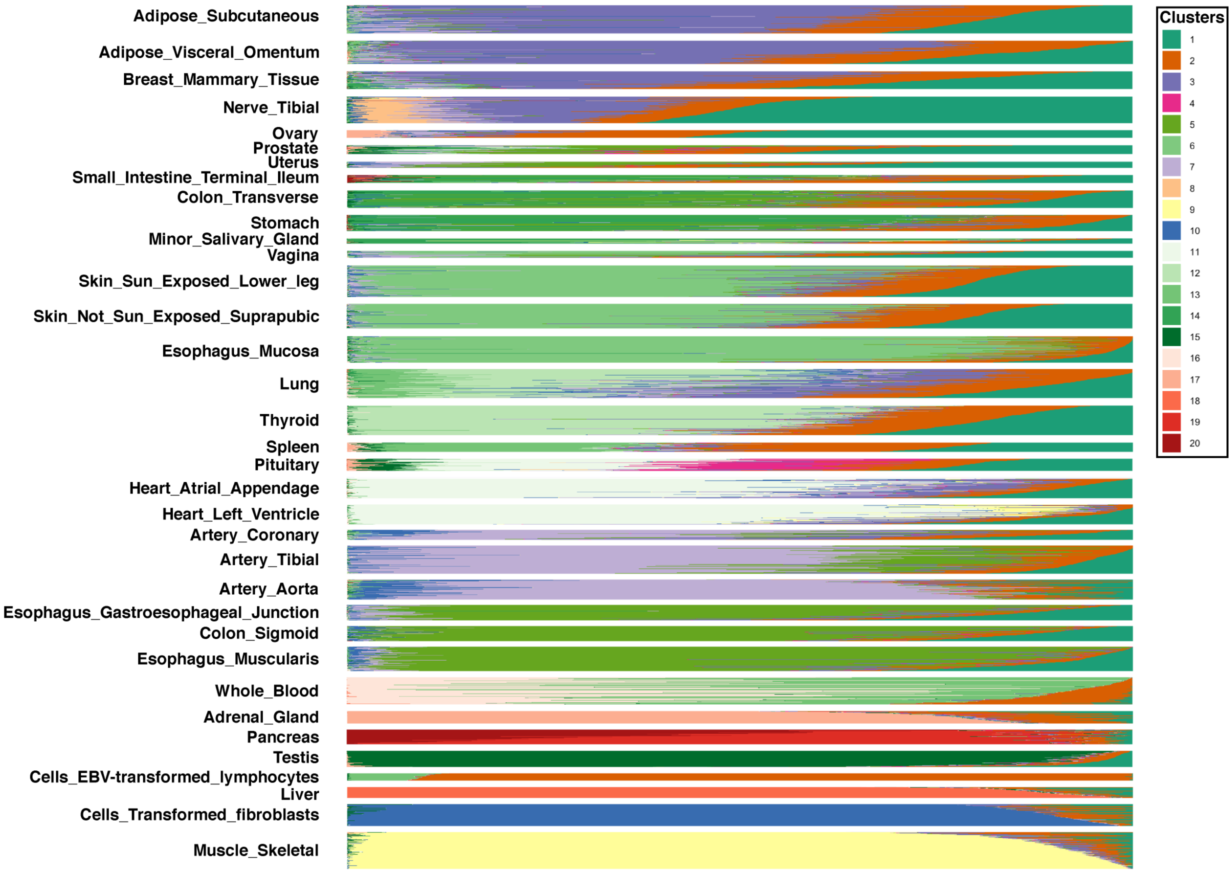

**Supplementary Figure 11. GTEx tissues heatmap plot.** Heatmap plot of estimated membership proportions using Grade of Membership model with K = 20 clusters. Model is fitted using 8,547 samples from 48 tissues in GTEx data.

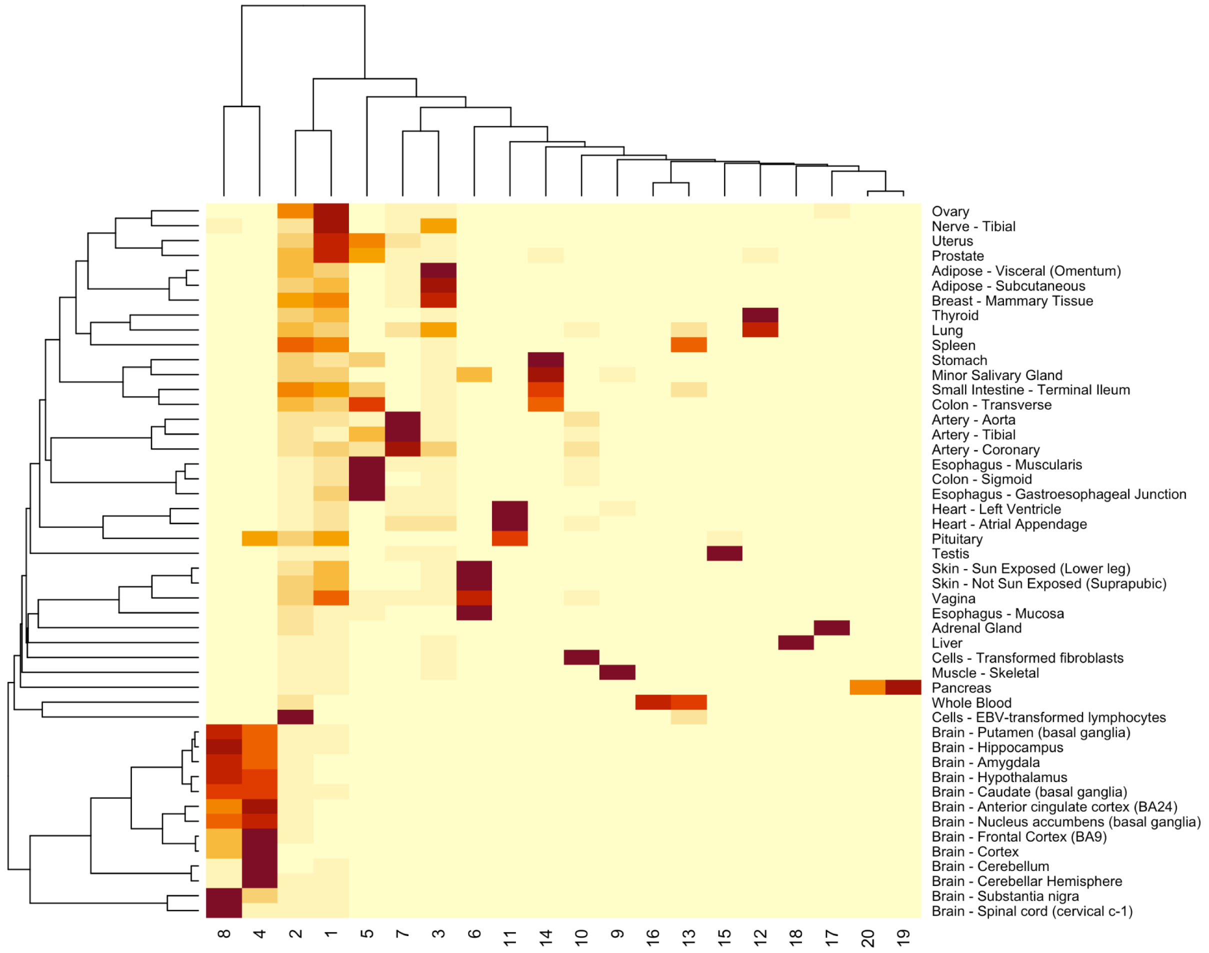

**Supplementary Figure 12. GTEx brain tissues structure and heatmap plots. a,** Structure plot of estimated membership proportions for grade of membership model with K = 6 clusters fit to 1,314 tissue samples from 13 brain tissues in GTEx data. Each horizontal bar represents the cluster membership proportions for a single sample. **b,** Heatmap plot of estimated membership proportions for grade of membership model with K = 6 clusters fit to 1,314 tissue samples from 13 brain tissues in GTEx data. Here, we fit the model to only brain tissues to explore the finer-scale structure among brain samples, which would be missed by the global analysis.

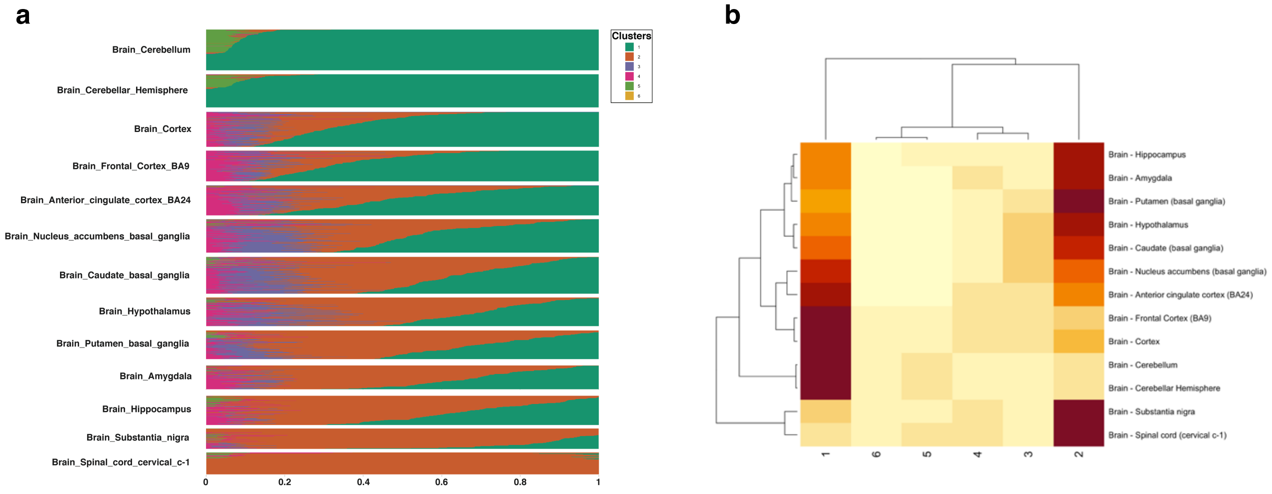

**Supplementary Figure 13. Principle component analysis of genetic data from different RNA-seq datasets.** We use 1000 Genome Project Phase 3 as a reference cohort and apply ADMIXTURE to determine the ancestry of each sample in the rest of the cohorts. We plot principal components (PCs) of samples from (**a**) 1000 Genomes Project, (**b**) GTEx cohort Project, (**c**) Common Mind Consortium study, and (**d**) Depression Gene Network study. Individuals with the same ADMIXTURE ancestry assignment are plotted with the same color. It is clear that on the PC map, individuals with the same ancestry assignment are clustered together, which verify the correctness of the assignment. We only kept individuals with European ancestry fraction > 0.9 (light blue dots) according to ADMIXTURE analysis.

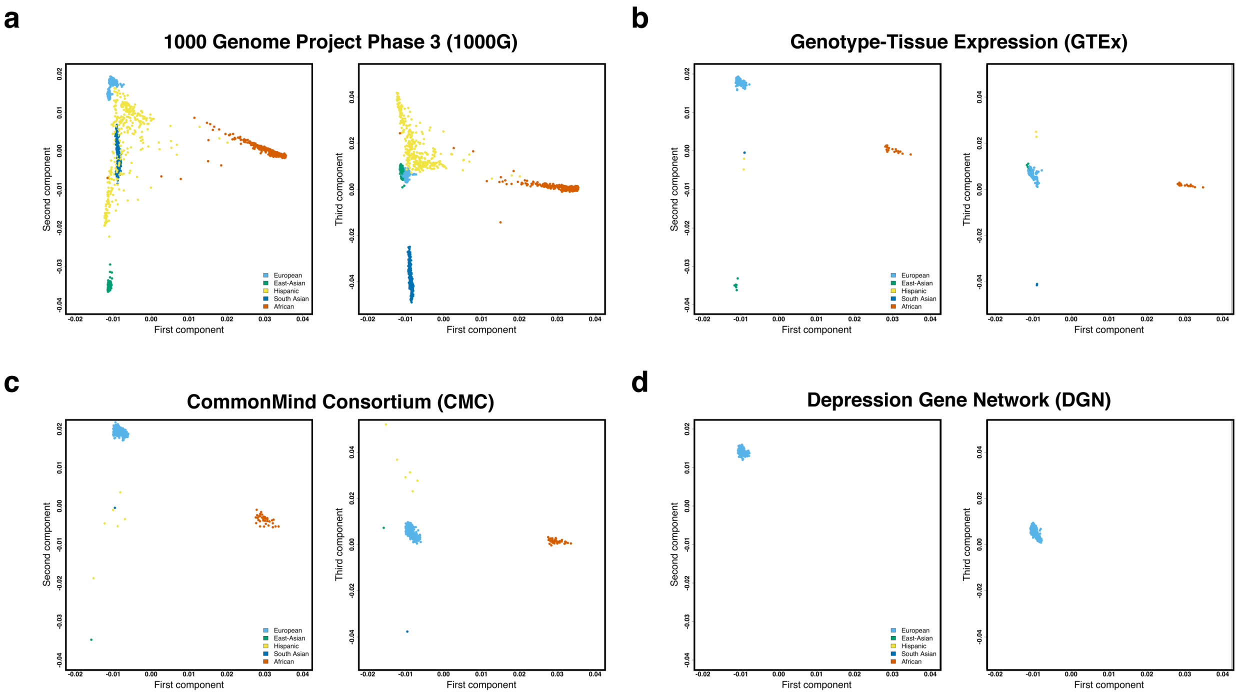

**Supplementary References**


1. The Genotype-Tissue Expression (GTEx) project. *Nat Genet* **45**, 580-585 (2013).
2. Battle A*, et al.* Characterizing the genetic basis of transcriptome diversity through RNA-sequencing of 922 individuals. *Genome Res* **24**, 14-24 (2014).
3. Fromer M*, et al.* Gene expression elucidates functional impact of polygenic risk for schizophrenia. *Nat Neurosci* **19**, 1442-1453 (2016).
4. Alexander DH, Lange K. Enhancements to the ADMIXTURE algorithm for individual ancestry estimation. *BMC Bioinformatics* **12**, 246 (2011).
5. Auton A*, et al.* A global reference for human genetic variation. *Nature* **526**, 68-74 (2015).
6. Lappalainen T*, et al.* Transcriptome and genome sequencing uncovers functional variation in humans. *Nature* **501**, 506-511 (2013).
7. Purcell S*, et al.* PLINK: a tool set for whole-genome association and population-based linkage analyses. *Am J Hum Genet* **81**, 559-575 (2007).
8. Gusev A*, et al.* Integrative approaches for large-scale transcriptome-wide association studies. *Nat Genet* **48**, 245-252 (2016).
9. Zhou X, Stephens M. Genome-wide efficient mixed-model analysis for association studies. *Nature Genetics* **44**, 821-824 (2012).
10. Zhou X, Carbonetto P, Stephens M. Polygenic Modeling with Bayesian Sparse Linear Mixed Models. *PLOS Genetics* **9**, e1003264 (2013).
11. Zeng P, Zhou X. Non-parametric genetic prediction of complex traits with latent Dirichlet process regression models. *Nature Communications* **8**, 456 (2017).
12. Nagpal S*, et al.* TIGAR: An Improved Bayesian Tool for Transcriptomic Data Imputation Enhances Gene Mapping of Complex Traits. *Am J Hum Genet* **105**, 258-266 (2019).
13. Zhang W*, et al.* Integrative transcriptome imputation reveals tissue-specific and shared biological mechanisms mediating susceptibility to complex traits. *Nat Commun* **10**, 3834 (2019).
14. Friedman JH, Hastie T, Tibshirani R. Regularization Paths for Generalized Linear Models via Coordinate Descent. *2010* **33**, 22 (2010).
15. Hu Y*, et al.* A statistical framework for cross-tissue transcriptome-wide association analysis. *Nat Genet* **51**, 568-576 (2019)
