# Supplementary material for: Integrating 3D genomic and epigenomic data to enhance target gene discovery and drug repurposing in transcriptome-wide association studies
Source: Nat Commun. 2022 Jun 7;13:3258. doi: 10.1038/s41467-022-30956-7 (PMC9171100; doi:10.1038/s41467-022-30956-7)
Supplement: Supplementary file 3 — Description of Additional Supplementary Files [file 41467_2022_30956_MOESM3_ESM.pdf]

Description of Additional Supplementary Files

File Name: **Supplementary Data 1**
Description: Type I error under null simulation studies at three significance levels: 0.05, 0.005, and 0.0005**.**

File Name: **Supplementary Data 2**
Description: Nested cross-validation prediction performance (median value of Spearman's correlation coefficient (r)) across 48 GTEx tissues. Here, we compared PUMICE to PrediXcan, FUSION, TIGAR, EpiXcan, and UTMOST, respectively.

File Name: **Supplementary Data 3**
Description: Number of significant models aross all TWAS methods. For EpiXcan, we trained models in 8 GTEx tissues due to the limited availability of EpiXcan models.

File Name: **Supplementary Data 4**
Description: Comparison of prediction performance in external datasets between PUMICE and other TWAS methods (i.e. PrediXcan, EpiXcan, FUSION, TIGAR, and UTMOST).

File Name: **Supplementary Data 5**
Description: Comparison of prediction performance in external datasets between PUMICE and EpiXcan. Here, we trained the model in GTEx Brain_Frontal_Cortex_BA9 tissue and predicted into the CMC dataset. We made sure to utilize similar ROADMAP epigenomic annotation used by EpiXcan.

File Name: **Supplementary Data 6**
Description: Comparison of prediction performance in external datasets between PUMICE and multi-tissue TWAS methods (i.e., UTMOST). Single tissue method can be applied to larger datasets in single tissue, which often yields much improved prediction accuarcy than multi-tissue methods that requires datasets with gene expression with multi-tissues measured. Here we compare prediction models by PUMICE trained in larger external single tissue data with the prediction model trained by UTMOST in GTEx. PUMICE substantially improves the prediction accuracy.

File Name: **Supplementary Data 7**
Description: Numbers of significant gene x trait associations (GTAs), Numbers of unique and independent significant gene x trait associations (Indep count), Numbers of independent and novel significant gene x trait associations (Novel count), and median chi-square values at MAGMA-prioritized genes across 3 GTEx tissues and 3 external datasets. It should be noted that UTMOST can only be trained in GTEx. We found that PUMICE identified more independent and novel gene x trait associations than that of UTMOST when trained in external datasets. In comparison to other single-tissue TWAS methods, we found that PUMICE"s performance became comparable to other methods when training sample size is extremely high (i.e., DGN at 873 samples).

File Name: **Supplementary Data 8**

Description: Distribution of the number of genes for which a given window size (i.e., 1000kb, 250kb, Domain, Loop, pcHiC and TAD) is chosen in PUMICE. These selected regions were used to derive gene expression prediction models (all significant genes).

File Name: **Supplementary Data 9**
Description: Distribution of PUMICE's selected penalty factor (for established predictors) used to derive gene expression prediction models (all significant genes).

File Name: **Supplementary Data 10**
Description: Information of 79 GWAS traits.

File Name: **Supplementary Data 11**
Description: Numbers of significant gene x trait associations across 48 tissues for 79 complex traits at all loci. Gene x trait associations identified in multiple tissues were separately counted.

File Name: **Supplementary Data 12**
Description: Numbers of unique significant gene x trait associations across 48 tissues for 79 complex traits at all loci. Gene x trait associations identified in multiple tissues were counted only once.

File Name: **Supplementary Data 13**
Description: Numbers of unique and independent significant gene x trait associations across 48 tissues for 79 complex traits at all loci. Here, we systemically pruned out significant genes that are in the vicinity (1 Mb window) of the most significant gene.

File Name: **Supplementary Data 14**
Description: Numbers of independent and novel significant gene x trait associations across 48 tissues for 79 complex traits at novel loci. Gene x trait association identified in different tissue types were counted only once. Here, known loci are defined by +/- 1Mb from sentinel variants.

File Name: **Supplementary Data 15**
Description: List of traits (23 in total) available for computational drug repurposing analysis.

File Name: **Supplementary Data 16**
Description: Results of compuational drug repurposing predictions across all TWAS methods. CMap tau scores are illustrated. These results correspond to the heatmap plots in Figure 6.

File Name: **Supplementary Data 17**
Description: Finemap results by FOCUS and our method. Here, we applied FOCUS and our fine-mapping method to TWAS results across 48 GTEx tissues and 79 GWAS traits. To compare our finemap result to FOCUS, we considered only the regions shared between the two methods and calculate the mean of credible set size. In general, we found that PUMICE and PUMICE+ typically led to the significantly smaller credible set size. Furthermore, we showed that 78% of the credible sets shared similarly finemapped genes.

File Name: **Supplementary Data 18**
Description: Sample sizes of 48 GTEx tissues. For each tissue type, total sample sizes and sample sizes of individuals with European ancestry (both self-reported and inferred by ADMIXTURE program) are listed. Data is sorted by the sample sizes of European sample sizes (as inferred by ADMIXTURE program) from smallest to largest.

File Name: **Supplementary Data 19**
Description: A list of available tissues and cell lines with 3D genomic data

File Name: **Supplementary Data 20**
Description: Matched tissues between GTEx and 3D genomic data via CountCluster. 3D genomic data is not available for all GTEx tissues. As a result, we chose 3D genomic data from proxy tissues and used them to help define regions that harbor regulatory variants. Proxy tissues were determined by similarities between expression profiles, as 3D genome organizational structures are strongly correlated with gene expression profiles and remain stable across similar tissue types.

File Name: **Supplementary Data 21**
Description: A list of available tissues and cell lines for epigenomics data (https://screen-v10.wenglab.org/)

File Name: **Supplementary Data 22**
Description: Number of genes in each tissue and corresponding TWAS Bonferroni-corrected p-value threshold (0.05/number of measured genes in each tissue).
