# Supplementary material for: Integrating 3D genomic and epigenomic data to enhance target gene discovery and drug repurposing in transcriptome-wide association studies
Source: Nat Commun. 2022 Jun 7;13:3258. doi: 10.1038/s41467-022-30956-7 (PMC9171100; doi:10.1038/s41467-022-30956-7)
Supplement: Supplementary file 5 — Reporting Summary [file 41467_2022_30956_MOESM5_ESM.pdf]

nature research

Corresponding author(s): Dajiang J. Liu, Bibo Jiang

Last updated by author(s): Apr 9, 2022

Reporting Summary

Nature Research wishes to improve the reproducibility of the work that we publish. This form provides structure for consistency and transparency in reporting. For further information on Nature Research policies, see our Editorial Policies and the Editorial Policy Checklist.

Statistics

For all statistical analyses, confirm that the following items are present in the figure legend, table legend, main text, or Methods section.

n/a	Confirmed	
☐	☒	The exact sample size (*n*) for each experimental group/condition, given as a discrete number and unit of measurement
☒	☐	A statement on whether measurements were taken from distinct samples or whether the same sample was measured repeatedly
☐	☒	The statistical test(s) used AND whether they are one- or two-sided<br>*Only common tests should be described solely by name; describe more complex techniques in the Methods section.*
☐	☒	A description of all covariates tested
☐	☒	A description of any assumptions or corrections, such as tests of normality and adjustment for multiple comparisons
☐	☒	A full description of the statistical parameters including central tendency (e.g. means) or other basic estimates (e.g. regression coefficient) AND variation (e.g. standard deviation) or associated estimates of uncertainty (e.g. confidence intervals)
☐	☒	For null hypothesis testing, the test statistic (e.g. *F*, *t*, *r*) with confidence intervals, effect sizes, degrees of freedom and *P* value noted<br>*Give P values as exact values whenever suitable.*
☒	☐	For Bayesian analysis, information on the choice of priors and Markov chain Monte Carlo settings
☒	☐	For hierarchical and complex designs, identification of the appropriate level for tests and full reporting of outcomes
☐	☒	Estimates of effect sizes (e.g. Cohen's *d*, Pearson's *r*), indicating how they were calculated

Our web collection on statistics for biologists contains articles on many of the points above.

Software and code

Policy information about availability of computer code

Data collection	No software was used for data collection.

Data analysis	PUMICE pipeline and implementation version 1.0.0, https://github.com/ckhunsr1/PUMICE (DOI: 10.5281/zenodo.6426359); PrediXcan software version 0.6.5, https://github.com/hakyimlab/MetaXcan (Code from PUMICE (adapted from PrediXcan) was used to run PrediXcan); FUSION software, https://github.com/gusevlab/fusion_twas (BLUP/BSLMM methods applied using gemma version 0.98.1); EpiXcan software, https://bitbucket.org/roussoslab/epixcan/src/master/ (Penalty factors were provided directly by EpiXcan author-Wen Zhang); TIGAR software, https://github.com/yanglab-emory/TIGAR (DPR methods applied using DPR software version 0.95alpha https://github.com/biostatpzeng/DPR); CTIMP (UTMOST) software, https://github.com/yiminghu/CTIMP; Genotype data processing, PLINK version 1.9 https://www.cog-genomics.org/plink/1.9/; Genotype imputation, Michigan Imputation Server https://imputationserver.sph.umich.edu/index.html#!; Ancestry estimation, ADMIXTURE version 1.3.0 https://bioinformaticshome.com/tools/descriptions/ADMIXTURE.html; Clustering RNA-seq expression data, CountClust package CountClust R package version 1.14.0; BED file processing, bedtools version 2.29.2 https://bedtools.readthedocs.io/en/latest/; Computational drug repurposing analysis, https://clue.io/repurposing-app; Other R packages, tidyr R package version 1.1.2; dplyr R package version 1.0.2; IRanges R package version 2.24.0; GenomicRanges R package version 1.42.0; glmnet R package version 4.0-2; tidyverse R package version 1.3.0; genefilter R package version 1.72.0; caret R package version 6.0-86; ggpubr R package version 0.4.0; ggforce R package version 0.3.2; reshape2 R package version 1.4.4; ggplot2 R package version 3.3.2; RColorBrewer R package version 1.1-2; ggrepel R package version 0.8.2; ggpattern R package version 0.1.3; colortools R package version 0.1.5; qqman R package version 0.1.4; lattice R package version 0.20-38; data.table R package version 1.13.0; matrixStats R package version 0.56.0; stringr R package version 1.4.0; plink2R R package version 1.1; rareGWAMA R package version 0.4; patchwork R package version 1.1.0.9000.

For manuscripts utilizing custom algorithms or software that are central to the research but not yet described in published literature, software must be made available to editors and reviewers. We strongly encourage code deposition in a community repository (e.g. GitHub). See the Nature Research guidelines for submitting code & software for further information.

Data

Policy information about availability of data

All manuscripts must include a data availability statement. This statement should provide the following information, where applicable:

- Accession codes, unique identifiers, or web links for publicly available datasets
- A list of figures that have associated raw data
- A description of any restrictions on data availability

Summary statistic files are publicly available and Pubmed ID unique identifiers associated with each study is provided in Supplementary Data 10. PUMICE Gene expression prediction models (trained in 48 tissues of GTEx v7) can be found at https://github.com/ckhunsr1/PUMICE/tree/master/models (DOI: 10.5281/zenodo.6426359).

Field-specific reporting

Please select the one below that is the best fit for your research. If you are not sure, read the appropriate sections before making your selection.

☒ Life sciences ☐ Behavioural & social sciences ☐ Ecological, evolutionary & environmental sciences

For a reference copy of the document with all sections, see nature.com/documents/nr-reporting-summary-flat.pdf

Life sciences study design

All studies must disclose on these points even when the disclosure is negative.

Sample size	We directly utilized normalized expression data from GTEx, which is the largest available multi-tissue gene expression dataset; therefore, we did not pre-determine sample size.
Data exclusions	Non-European samples were excluded from this study since the sample sizes of non-European ancestries are small.
Replication	Gene expression prediction models were validated in three external datasets. Specifically, we validated the performance of gene expression prediction models in three GTEx tissues with matching external datasets (i.e. whole blood from GTEx and Depression Gene Network, brain frontal cortex BA9 from GTEx and Common Mind Consortium, and lymphoblastoid cell lines from GTEx and Genetic European Variation in Disease).
Randomization	Randomization is not applicable since we used published datasets. No new data was collected.
Blinding	The data were publicly available; therefore, the investigators were not blinded to group allocations.

Reporting for specific materials, systems and methods

We require information from authors about some types of materials, experimental systems and methods used in many studies. Here, indicate whether each material, system or method listed is relevant to your study. If you are not sure if a list item applies to your research, read the appropriate section before selecting a response.

Materials & experimental systems

n/a	Involved in the study
☒	☐ Antibodies
☒	☐ Eukaryotic cell lines
☒	☐ Palaeontology and archaeology
☒	☐ Animals and other organisms
☒	☐ Human research participants
☒	☐ Clinical data
☒	☐ Dual use research of concern

Methods

n/a	Involved in the study
☒	☐ ChIP-seq
☒	☐ Flow cytometry
☒	☐ MRI-based neuroimaging
